# NUP62 localizes to ALS/FTLD pathological assemblies and contributes to TDP-43 insolubility

**DOI:** 10.1038/s41467-022-31098-6

**Published:** 2022-06-13

**Authors:** Amanda M. Gleixner, Brandie Morris Verdone, Charlton G. Otte, Eric N. Anderson, Nandini Ramesh, Olivia R. Shapiro, Jenna R. Gale, Jocelyn C. Mauna, Jacob R. Mann, Katie E. Copley, Elizabeth L. Daley, Juan A. Ortega, Maria Elena Cicardi, Evangelos Kiskinis, Julia Kofler, Udai B. Pandey, Davide Trotti, Christopher J. Donnelly

**Affiliations:** 1 Department of Neurobiology, University of Pittsburgh School of Medicine, Pittsburgh, PA USA; 2 LiveLikeLou Center for ALS Research, University of Pittsburgh Brain Institute, Pittsburgh, PA USA; 3 Department of Neuroscience, Jefferson Weinberg ALS Center, Vickie and Jack Farber Institute for Neuroscience, Thomas Jefferson University, Philadelphia, PA USA; 4 Physician Scientist Training Program, University of Pittsburgh School of Medicine, Pittsburgh, PA USA; 5 Department of Pediatrics, Children’s Hospital of Pittsburgh, University of Pittsburgh Medical Center, Pittsburgh, PA USA; 6 Department of Human Genetics, University of Pittsburgh Graduate School of Public Health, Pittsburgh, PA USA; 7 Center for Neuroscience, University of Pittsburgh, Pittsburgh, PA USA; 8 The Ken & Ruth Davee Department of Neurology, Northwestern University of Feinberg School of Medicine, Chicago, IL USA; 9 Department of Neuroscience, Northwestern University Feinberg School of Medicine, Chicago, IL USA; 10 Department of Pathology, University of Pittsburgh, Pittsburgh, PA USA

**Keywords:** Mechanisms of disease, Amyotrophic lateral sclerosis

## Abstract

A G4C2 hexanucleotide repeat expansion in the *C9orf72* gene is the most common genetic cause of ALS and FTLD (C9-ALS/FTLD) with cytoplasmic TDP-43 inclusions observed in regions of neurodegeneration. The accumulation of repetitive RNAs and dipeptide repeat protein (DPR) are two proposed mechanisms of toxicity in C9-ALS/FTLD and linked to impaired nucleocytoplasmic transport. Nucleocytoplasmic transport is regulated by the phenylalanine-glycine nucleoporins (FG nups) that comprise the nuclear pore complex (NPC) permeability barrier. However, the relationship between FG nups and TDP-43 pathology remains elusive. Our studies show that nuclear depletion and cytoplasmic mislocalization of one FG nup, NUP62, is linked to TDP-43 mislocalization in C9-ALS/FTLD iPSC neurons. Poly-glycine arginine (GR) DPR accumulation initiates the formation of cytoplasmic RNA granules that recruit NUP62 and TDP-43. Cytoplasmic NUP62 and TDP-43 interactions promotes their insolubility and NUP62:TDP-43 inclusions are frequently found in C9orf72 ALS/FTLD as well as sporadic ALS/FTLD postmortem CNS tissue. Our findings indicate NUP62 cytoplasmic mislocalization contributes to TDP-43 proteinopathy in ALS/FTLD.

## Abstract (web summary)

ALS and FTLD are both characterized by insoluble cytoplasmic depositions of TDP43. Here the authors show that the nucleopore protein NUP62 is mislocalized in C9orf72 and sporadic ALS/FTLD and propose that it interacts with TDP-43 to promote its insolubility.

## Introduction

ALS and FTLD are fatal neurodegenerative disorders that exist along a disease spectrum with shared causative mutations and aberrant RNA-binding protein (RBP) mislocalization and inclusion formation^1,2^. TDP-43 and FUS are predominantly nuclear RBPs that cycle between the nucleus and cytoplasm and primarily regulate RNA metabolism^3^. However, these proteins are mislocalized to the cytoplasm and form neuropathological inclusions in 97% of ALS patients and up to half of FTLD patients^4–9^. In addition to shared neuropathology, the most common genetic cause of both ALS and FTLD is an expanded G4C2 hexanucleotide repeat sequence in the first intron of the *C9orf72* gene (C9-ALS/FTLD)^10–13^. The pathobiology underlying this mutation includes C9orf72 haploinsufficiency, the deposition of toxic repetitive RNAs, and cellular accumulation of dipeptide repeats (DPRs)^10,14–20^.

While the causative mechanisms driving C9-ALS/FTLD pathobiology remains unclear, significant work indicates that C9orf72 haploinsufficiency alone is not sufficient to initiate motor or cognitive phenotypes in rodent models^21^. However, complete loss of both alleles causes mild motor impairment as shown by reduced activity on the open-field test in C9-ALS/FTLD mouse models^22–24^. In contrast, expression of an expanded G4C2 sequence drives neurotoxicity in vitro^25,26^ and cognitive phenotypes in vivo in AAV and BAC transgenic rodent models^23,27–30^. Further, C9orf72 protein knockdown in two G4C2 repeat expansion mouse models caused a synergistic increase in cognitive defects and neurotoxicity to support the convergence of loss- and gain-of-function mechanisms of toxicity^31,32^.

C9-ALS/FTLD gain-of-function toxicity from the G4C2 expansion includes RNA:protein accumulations that manifest as RNA foci^10,33^, sequestering RBPs and impairing their normal function^15,33–39^. Furthermore, expanded repeat RNAs are translated into five DPRs from both G4C2 sense and C4G2 antisense strands through the non-canonical repeat-associated non-ATG translation (RANT) pathway^40–42^. These DPRs include glycine-arginine (GR), glycine-alanine (GA), proline-arginine (PR), proline-alanine (PA), and glycine-proline (GP)^43–46^. Both RNA foci and DPRs are found in patient-derived C9-ALS/FTLD iPSC-derived neuronal cultures^15,16,47^ and post-mortem tissue samples^10,33,48,49^. While it is difficult to distinguish between exclusively RNA-mediated toxicity and that of DPRs, both products are thought to contribute to C9-ALS/FTLD. Expression of codon-optimized sequences encoding DPRs allows us to understand DPR-specific effects and has been shown to cause excitotoxicity in neurons, abnormal nucleolar and mitochondrial functions, altered SG dynamics, ribosomal dysfunction, and cytotoxicity^50–59^. Notably, the most highlighted cellular process hypothesized to be disrupted in C9-ALS/FTLD and linked to these gain-of-function mechanisms is the nucleocytoplasmic (nuc/cyto) transport pathway^39,60–66^.

Nuc/cyto transport refers to the trafficking of proteins and RNAs across the nuclear membrane through the NPC^67^. The NPC is a large multi-subunit protein complex comprised of approximately 30 different protein subunits, known as nucleoporins or nups^68^. Nups serve a variety of functions but their most well-defined role involves creating the NPC permeability and selectivity barrier^69–71^. Molecules smaller than ~40 kDa can freely diffuse across the NPC^72^ whereas facilitated nuc/cyto transport of larger molecules is driven by a gradient of Ran:GTP^73–77^, and larger molecules are actively escorted through the NPC by nuclear transport receptors, or karyopherins. Karyopherins traverse the pore by directly interacting with FG nucleoporins (FG nups)^78–82^. FG nups make up approximately one-third of the nucleoporins and contain protein domains that are enriched in phenylalanine (F)-glycine (G) residues and associated with structural disorder and flexibility^83,84^. This enrichment of phenylalanine and glycine creates intrinsically disordered regions (IDRs) within FG nups that, in turn, contribute to the NPC permeability barrier by forming a hydrogel-like structure through liquid-liquid phase separation^85,86^.

The impact of G4C2 repeat expansion expression on nuc/cyto trafficking is well documented in a variety of model systems. Initial studies employing RNAi and chromosomal deletion genetic screens to identify modifiers of UAS-(G4C2)_30_ repeat and UAS-(G4C2)_58_ repeat toxicity in *Drosophila* retinal neurons (GMR-GAL4 driven expression) revealed several genes within the nuc/cyto transport pathway and NPC, such as FG nups, as potent modifiers of toxicity^60,64^. Furthermore, karyopherin overexpression was identified as a strong suppressor of PR_50_ toxicity in a yeast genetic screen^63^. Similarly, downregulation of several karyopherins and Ran gradient regulators enhanced degenerative eye phenotype in a PR_25_-expressing *Drosophila* model^62^ and GR_50_-expressing Drosophila^54^. In addition to genetic screens using invertebrate model systems, nuc/cyto trafficking dynamics are altered in C9-ALS/FTLD iPSC-derived neurons and regulators of transport were subsequently shown to exhibit abnormal staining in postmortem tissue^60,61,87,88^. Thus, the underlying premise is that NPC perturbations promote the TDP-43 mislocalization via nuc/cyto trafficking impairment. However, this does not explain the lack of other nuclear proteins one would suspect to be mislocalized if nuclear-cytoplasmic transport was disrupted. Thus, the mechanisms linking FG nups to cellular dysfunction and TDP-43 neuropathology remain undefined.

Here we examined the relationship between C9-ALS/FTLD and FG nups using multiple in vitro and in vivo model systems. Our studies focus on NUP62 since it is a critical FG nup with an extensive FG-repeat domain, located within the NPC central channel, and functions to regulates transport through the NPC^89–92^. We show that NUP62 is lost from the nucleus and abnormally localized to the cytoplasm in C9-ALS/FTLD model systems. Cytoplasmic NUP62 assemblies are formed in vitro and in vivo in response to GR_50_ expression. GR_50_ expression results in the formation of cytoplasmic condensates that contain TDP-43, RNA, stress granule proteins, and NUP62. Cytoplasmic NUP62 and TDP-43 interactions promote soluble-to-insoluble transition of these assemblies that appear dependent on the TDP-43 nuclear localization sequence (NLS). Consistent with this mechanism, Nup62 is a genetic modifier of neurotoxicity in C9-ALS/FTLD *Drosophila* models. We validated these findings through neuropathological analyses of post-mortem tissue and found NUP62 colocalizes with phosphorylated TDP-43 in C9-ALS/FTLD tissue. Interestingly, NUP62:phosphorylated-TDP-43 inclusions are also found in sporadic ALS/FTLD CNS tissue suggestive of a pathogenic role for NUP62 mislocalization in disease through interaction with TDP-43.

## Results

### NUP62 and TDP-43 are mislocalized in C9-ALS/FTLD

**Fig. 1:**
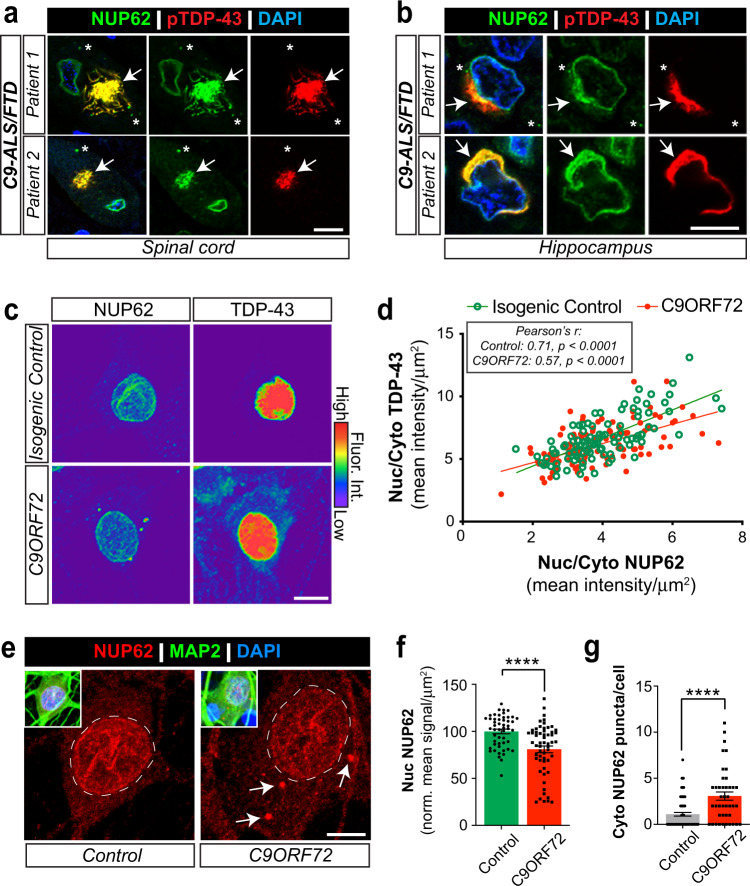
Cytoplasmic NUP62 is associated with TDP-43 mislocalization. **a**, **b** NUP62 (green) and phosphoTDP-43 (pTDP-43, red) immunoreactivity in postmortem tissue from C9-ALS/FTD clinical diagnoses. Cytoplasmic NUP62 is colocalized with (arrows) and without (asterisks) phosphoTDP-43. Colocalization of NUP62 and phosphoTDP-43 accumulations were observed in the spinal cord (**a**) and hippocampus (**b**). Patient diagnostic classifications are described in Supplemental Table 1. Scale bar: 10 µm (**c**) NUP62 and TDP-43 immunostaining in isogenic control (top row) and C9-ALS (bottom row) iPSC neurons (Supplemental Table 2) presented in spectral colors. Warmer colors represent higher NUP62 or TDP-43 levels while cooler colors show lower levels. Cytoplasmic NUP62 is linked with higher TDP-43 mislocalization in neurons. Scale bar: 12.5 µm. **d** Nuclear-cytoplasmic (Nuc/Cyto) distribution of NUP62 and TDP-43 were determined for isogenic control and C9-ALS iPSC neurons from maximum intensity projection confocal images. Values were then plotted for each individual neuron and simple linear regression was calculated to determine best fit-line as shown. Pearson’s Correlation Analysis was conducted. There was a positive correlation between Nuc/Cyto NUP62 and TDP-43 in isogenic control [r(101) = .71, *p* = 3.21e-017] and C9-ALS [r(100) = 0.57, *p* = 2.54e-010] neurons. **e** NUP62 immunostaining (red) is shown in the representative confocal maximum intensity projection images of healthy control and C9-ALS MAP2^+^ iPSC neurons that had been differentiated and matured for 89 days. DAPI^+^ nuclear compartment is highlighted with dashed white line and cytoplasmic NUP62 puncta are indicated by white arrows. Scale bar: 10 µm. **f** Quantification of nuclear NUP62 intensity shows lower levels in C9-ALS iPSC neurons. Average signal is shown by graph bars while dots and squares represent the signal in individual neurons. The control group (*n* = 59 neurons) consists of two separate iPSC lines and C9ORF72 (*n* = 58 neurons) is the combination of three C9-ALS iPSC lines. Data are shown as mean + /- SEM. **g** The number of NUP62 puncta in MAP2^+^ iPSC neurons is quantified from confocal images represented in Fig. 1e. Data analysis reveals an increase in cytoplasmic NUP62 puncta quantity in C9-ALS iPSC neurons. Average cytoplasmic NUP62 puncta per cell are show by the graph bars while individual cell data are shown by the dots and squares (*n* = 70 (control) or 42 (C9ORF72) neurons). Data are shown as mean + /- SEM. Statistical significance was determined by unpaired, two-tailed student’s *t*-test. **** *p* ≤ 0.0001 vs control in **f** & **g**.

In vitro expression systems previously showed that nucleoporins can abnormally localize to the cytoplasm through interactions with the TDP-43 C-terminal fragment^93^. Therefore, we conducted neuropathological analysis of C9-ALS/FTLD patient postmortem CNS tissue to determine whether phosphorylated TDP-43 inclusions correlated with abnormal NUP62 signal. In addition to nuclear membrane staining, we observed cytoplasmic NUP62 to be present in spinal cord and hippocampal post-mortem tissue by immunohistochemistry (Fig. 1a, b, arrows and asterisks, Supplementary Table 6). Interestingly, NUP62 staining frequently colocalized with phospho-TDP-43^+^ (pTDP-43) inclusions in neurons of the spinal cord and hippocampus of C9-ALS/FTLD cases (Fig. 1a, b, arrow), suggesting a link between TDP-43 inclusions and cytoplasmic NUP62 mislocalization.

TDP-43 was previously shown to be modestly enriched in the cytoplasm of C9-ALS/FTLD iPSC neurons^60^, which also observed in this study (Supplementary Figure 1a, b). Therefore, we investigated whether TDP-43 mislocalization correlated with cytoplasmic NUP62 in C9-ALS/FTLD iPSC motor neurons. We performed NUP62 and TDP-43 immunofluorescent staining in isogenic control and C9-ALS/FTLD iPSC neurons and acquired images by confocal microscopy (Fig. 1c). For each MAP2^+^ cell, we plotted nuclear-cytoplasmic TDP-43 mean intensity (Y-axis) and nuclear-cytoplasmic NUP62 mean intensity (X-axis) (Fig. 1d). We then conducted Pearson’s correlation analysis to examine the relationship between nuc/cyto TDP-43 and nuc/cyto NUP62 distribution. We found a modest, but statistically significant, Pearson’s correlation for both isogenic control (Pearson’s correlation coefficient r: 0.71) and C9-ALS/FTLD (Pearson’s correlation coefficient r: 0.58) iPSC neurons (Fig. 1d). This suggests that the localization of cytoplasmic TDP-43 correlates with cytoplasmic NUP62 in both C9-ALS/FTLD iPSC neurons (with enriched cytoplasmic TDP-43) and isogenic controls. In contrast, there is no correlation between nuclear NUP62 and nuc/cyto TDP-43 in these cells (Supplementary Figure 1c). Together, these data strongly indicate a relationship between cytoplasmic NUP62 and cytoplasmic TDP-43.

We next tested whether nuclear NUP62 loss and cytoplasmic accumulation is observed in C9-ALS/FTLD iPSC motor neurons. Differentiated control and C9-ALS/FTLD iPSC motor neuron cultures (2 control lines & 3 C9-ALS/FTLD lines; Supplementary Table 1) were assessed at 89 days post-differentiation^39^ and immunostained for NUP62 and MAP2 (Fig. 1e). Quantification of maximum intensity projection confocal images revealed a 19.1% reduction in nuclear NUP62 (Fig. 1f) and a 279% enrichment of cytoplasmic NUP62 in C9-ALS/FTD iPSC neurons compared to controls (Fig. 1e-g, white arrows) (Control: 1.1 vs C9-ALS/FTD: 3.07 puncta/MAP2^+^ neuron). Together, these data indicate that NUP62 is mislocalized in vivo and in C9-ALS/FTD iPSC-derived neurons in vitro.

### Poly-GR sequesters TDP-43 and NUP62 through cytoplasmic RNA granules

**Fig. 2:**
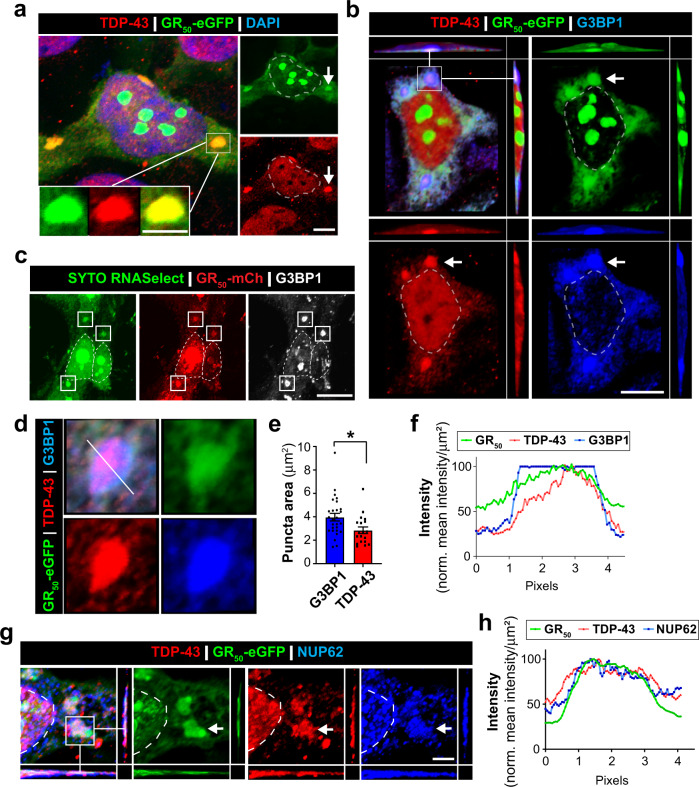
Poly-GR induces RNA granules that recruit TDP-43 and NUP62. HEK293 cells were transfected with GR_50_-eGFP plasmid DNA and immunostained prior to imaging by confocal microscopy. Images reveal the accumulation of cytoplasmic GR_50_-eGFP condensates that we went on to characterize. **a** Maximum intensity projection image reveals endogenous TDP-43 and GR_50_-eGFP localize together in cytoplasmic condensates. Scale bar: 10 μm. Inset image highlights a single condensate containing GR_50_-eGFP and endogenous TDP-43. Scale bar: 5 μm. **b** Orthogonal view of cytoplasmic GR_50_-eGFP condensates in HEK293 cells reveals endogenous TDP-43 and SG marker G3BP1 exist within same three-dimensional space. Top left panel is a merged image and others are of individual channels. Scale bar: 10 μm. **c** The presence of RNA in GR_50_-mCh condensates was determined with SYTO RNASelect green-fluorescent stain. Areas of intense fluorescent staining coincided with GR_50_-mCh^+^ and G3BP1^+^ structures. Thus, indicating the presence of RNA in GR_50_ condensates. White box highlights condensates. Scale bar: 10 μm. **d** Cropped image of cytoplasmic GR_50_-eGFP accumulations (from subpanel B) that we observe in cells. Immunocytochemistry for endogenous TDP-43 and G3BP1 reveal these proteins are detectable in the GR_50_-eGFP condensates. **e** G3BP1 (*n* = 27) and TDP-43 (*n* = 20) surface area was measured in GR_50_-eGFP^+^ condensates. We observe that TDP-43 is significantly smaller than G3BP1 in these GR_50_-eGFP structures. Data are shown as mean + /- SEM. Statistical significance was determined by unpaired two-tailed student’s t-test. * *p* ≤ 0.05 G3BP1 vs TDP-43. **f** Intensity profile plots for condensates containing GR_50_-eGFP, TDP-43, and G3BP1. An intensity profile plot line was drawn through the condensate and signal intensity is plotted across the length of line. This data further supports the hypothesis that these proteins exist within the same space. **g** Orthogonal view of cytoplasmic GR50-eGFP condensates in HEK293 cells reveals endogenous TDP-43 and NUP62 exist within same three-dimensional space. Left panel is a merged image of all signals together and the other panels are of individual channels. Scale bar: 5 µm. **h** Intensity profile plots for condensate containing GR_50_-eGFP, TDP-43 and NUP62. An intensity profile plot line was drawn through the condensate and signal intensity is plotted across the length of line. This data further supports the hypothesis that these proteins exist within the same space. Scale bar: 5 µm.

The cytoplasmic mislocalization and phosphorylation of TDP-43 (pTDP-43) is a pathological hallmark observed in ALS/FTLD patients^4,8^. Recent neuropathological studies show that while ~4% of TDP-43 inclusions contain GR in C9orf72 ALS/FTLD post-mortem tissue, most poly-GR accumulations colocalize with pTDP43^94^. Therefore, we tested whether GR_50_ expression promotes TDP-43 mislocalization. HEK293 cells were transfected with eGFP-tagged GR_50_ constructs with 50 glycine-arginine repeats (GR_50_-eGFP). Cells expressing GR_50_-eGFP exhibit cytoplasmic mislocalization of endogenous TDP-43 as observed by immunostaining and nuclear/cytoplasmic ratio analyses (Supplementary Figure 2a & b). Interestingly, GR_50_-eGFP-expressing cells formed droplet-like cytoplasmic condensates that colocalized with endogenous TDP-43 (Fig. 2a). Analyses of these cytoplasmic GR_50_:TDP-43 structures revealed high colocalization with a Pearson’s coefficient of 0.7233 (p-value: 0.0015; Fig. 2a, inset). Consistent with this, orthogonal renderings showed that cytoplasmic GR_50_-eGFP and TDP-43 exist together within the same three-dimensional space (Fig. 2a, b). Notably, this indicates that cytoplasmic GR depositions are sufficient to promote the formation of endogenous TDP-43 cytoplasmic condensates. This is supported by recent findings that show poly-GR drives aberrant phase-separation of purified or overexpressed TDP-43^95^ and these data indicate that endogenous TDP-43 similarly interacts with poly-GR inclusions in vitro.

Multiple cellular pathways are thought to contribute to aberrant TDP-43 phase separation and resulting pathological inclusions. Altered dynamics in membraneless organelles formed through liquid-liquid phase separation, such as SGs, are hypothesized to promote TDP-43 proteinopathy due to genetic mutations and altered composition. This is supported by work showing that chronic SG induction can promote cytoplasmic phosphorylated TDP-43 species in some cell lines^96^. Recent studies also identified SG-independent mechanisms that promote aberrant TDP-43 phase transitions, aggregation, and nuclear loss^97–99^. Furthermore, GR_50_ interactome includes TDP-43, SG proteins, and NPC components^54^. Therefore, we tested whether the GR:TDP-43 condensates resemble SG by immunostaining for G3BP1 and ATAXIN-2. Notably, cytoplasmic GR condensates colocalize with G3BP1 and ATAXIN-2 (Fig. 2b, Supplementary Figure 2c). These assemblies contain RNA, a characteristic of a functional SG^100^, as assessed by SYTO RNASelect fluorescent staining in GR_50_-mCh^+^ expressing cells (Fig. 2c). These data suggest that cytoplasmic GR condensates are RNA granules that contain SG proteins. GR_50_-induction of ATAXIN-2 assemblies is concentration-dependent, requiring high cellular levels of GR_50_-eGFP to form SGs (Supplementary Figure 2c). Analysis of G3BP1 and TDP-43 in the accumulated structures revealed TDP-43 surface area is significantly less than that of G3BP1, further supporting a SG-like nature (Fig. 2d & e). We then performed intensity profile plot analysis to understand protein localization within the structure and observed consistent TDP-43 localization within the GR and G3BP1 condensates to indicate their colocalization in vitro (Fig. 2f and Supplementary Figure 2d-e). We next characterized whether GR promotes cytoplasmic NUP62 localization through the formation of aberrant RNA granules and found NUP62 is sequestered into cytoplasmic GR-induced SG-like structures along with TDP-43 and ATAXIN-2 (Fig. 2g, Supplementary Figure 2c, f). Profile plot analysis of cytoplasmic GR structures revealed NUP62 within GR_50_-eGFP:TDP-43 condensates (Fig. 2h, Supplementary Figure 2g-h). Various nucleoporins have been previously identified as being colocalized with poly-GR^61,95^. Therefore, we tested whether other nucleoporins are present in GR-G3BP1 condensates by immunostaining and found NUP54, NUP98, and NUP153 within GR_50_:G3BP1^+^ condensates following GR_50_-mCh transfection in HEK293 cells (Supplementary Figure 3a-c). Together, these data indicate that cellular GR_50_ deposition induces the formation of SG-like, RNA granules structures that recruit endogenous TDP-43 and NUP62 to the cytoplasm.

### Glycine-arginine drives cytoplasmic NUP62 mislocalization in vitro and in vivo

**Fig. 3:**
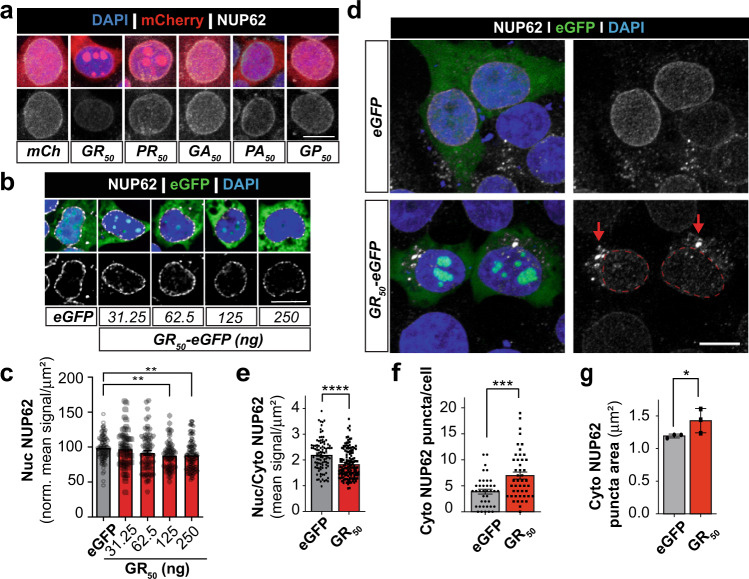
Poly-GR alters NUP62 localization in vitro. **a** Nuclear NUP62 (white) levels were assessed in HEK293 cells expressing mCherry-tagged poly-DPR constructs (red) by immunofluorescent staining and confocal microscopy. Representative maximum intensity projection images are shown. Nuclear NUP62 quantification is shown in Supplemental Fig. 4a. Scale bar: 10 µm. **b** HEK293 cells were transfected with increasing amounts of GR_50_-eGFP plasmid DNA and immunostained for NUP62. Image were processed by automatic deconvolution in Nikon Elements. Single slice images (0.2 µm) of NUP62 (white) show dose-dependent reduction in nuclear NUP62 with increasing GR_50_-eGFP plasmid DNA. Scale bar: 10 µm **c** Nuclear NUP62 signal was quantified in maximum intensity projection confocal images following immunofluorescent staining (*n* = 104 (eGFP), 62 (31.25 ng), 71 (62.5 ng), 64 (125 ng), 120 (250 ng) cells per group). Data corresponds to images presented in Fig. 3b. Statistical significance was determined by one-way ANOVA with Dunnett’s multiple comparisons test. Data are shown as mean + /- SEM. **d** To assess whether GR_50_-eGFP alters NUP62 localization, HEK293 cells were transfected with eGFP or GR_50_-eGFP (green) and immunostained for NUP62 (white). Representative confocal images show nuclear NUP62 depletion that coincides with cytoplasmic NUP62 puncta accumulation. Nuclear compartment is highlighted by dashed red line and cytoplasmic NUP62 accumulations are indicated by red arrow. Scale bar: 10 µm. **e** Quantification of nuclear/cytoplasmic (Nuc/Cyto) distribution of NUP62 signal corresponding to Fig. 3d representative images. Regions of interest (ROIs) were drawn around DAPI or cytoplasm signals to determine NUP62 in the respective regions. The distribution for each cell was determined and then averaged for *n* = 96 (eGFP) -138 (GR_50_) cells across four independent experiments. Statistical significance was determined by unpaired two-tailed t-test. Data are shown as mean + /- SEM. **f** We identified cytoplasmic NUP62 puncta by spot detection (diameter 1 µm or larger) methods and counted the frequency of these structures in each HEK293 cell (n = 39 (eGFP) - 47 (GR_50_) cells). Quantification reveals an increased cytoplasmic NUP62 prevalence due to GR_50_ expression. Statistical significance was determined by unpaired two-tailed t-test. Data are shown as mean + /- SEM. **g** Cytoplasmic NUP62 puncta were detected by ROI automatic detection and quantification reveals GR_50_-eGFP causes a significant increase in their size relative to eGFP control (n = 88 (eGFP) -117 (GR_50_) puncta that were evaluated over three biologically independent experiments). Statistical significance was determined by one-tailed, unpaired t-test. Data are shown as mean + /- SD. *p ≤ 0.05; **p ≤ 0.01; ***p ≤ 0.001; ****p ≤ 0.0001 vs eGFP control.

Irregularities in nucleoporin immunostaining were previously described in several C9-ALS/FTLD models but the direct relationship between late stage pathology (DPRs and TDP-43 mislocalization) and FG nups has not been examined in depth^60,64,88^. Our data indicate that cytoplasmic GR_50_ condensates promote NUP62 and TDP-43 mislocalization (Figs. 1 & 2). However, we do not yet know if poly-GR is the only DPR capable of disrupting NUP62 localization given that poly-PR has been shown to bind the FG domain of NUP54 and NUP98^65^. To test this, we expressed mCherry (mCh)-tagged poly-DPR constructs expressing 50 repeats of GR, PR, GA, PA, or GP^55^ in HEK293 cells and performed immunostaining and quantitative analyses of NUP62 via confocal microscopy. Our data showed that PR_50_, GA_50_, PA_50_, and GP_50_ do not alter nuclear NUP62 signal (Supplementary Figure 4a). However, GR_50_ significantly reduces nuclear NUP62 intensity by 15.25% after 24 h as compared to the mCh control (Fig. 3a, Supplementary Figure 4a). Furthermore, increasing concentrations of GR_50_-plasmid DNA transfected into HEK293 cells yields a greater reduction in nuclear NUP62 signal intensity (Fig. 3b, c) indicating a dose-dependent relationship between cellular GR_50_ burden and nuclear NUP62 loss. Furthermore, we find that control iPSC neurons with lentiviral-mediated GR_50_ expression exhibit significant depletion of nuclear NUP62 (Supplementary Figure 4b-c). Since nuclear envelope disruption is a common event during programmed cell death^101^ and poly-GR accumulation is cytotoxic in human cell lines and rodent models^55,56,102–105^, we quantified LDH release to determine whether nuclear NUP62 signal loss was due to GR_50_-mediated programmed cell death. No measurable difference was observed in DPR_50_ expressing cells at the time of fixation (Supplementary Figure 4d). This indicates that nuclear NUP62 depletion is associated with increasing cellular burden of GR. NUP62 is a short-lived nucleoporin and undergoes continual turnover^106,107^. Therefore, we tested whether GR_50_-mediated nuclear NUP62 deficits were due to transcriptional loss through RT-qPCR and found no significant difference between NUP62 mRNA levels in eGFP- and GR_50_- transfected cells after 24 h (Supplementary Figure 4e). This indicates NUP62 defects are due to increasing cytoplasmic GR_50_ burden and not transcriptional disruption.

We next examined whether GR_50_ promotes the cytoplasmic mislocalization of NUP62 protein. GR_50_-eGFP was expressed in HEK293 cells and nuclear/cytoplasmic NUP62 signals were quantified following immunostaining and confocal imaging. GR_50_-eGFP expression causes a modest but significant reduction in the nuc/cyto ratio of NUP62 protein, indicating an enhanced relative cytoplasmic NUP62 signal (Fig. 3d & e). We also assessed whether expression of a different DPR, GA_50_-eGFP, expression in HEK293 causes abnormal NUP62 localization. Although GA_50_-eGFP forms areas of condensed eGFP signal like GR_50_, NUP62 nuc/cyto distribution is not different from control (eGFP-expressing) HEK293 cells (Supplementary Figure 4f-g). Consistent with this, GR_50_ expression produced cytoplasmic NUP62 puncta that appeared more frequently (Fig. 3d & f) and with a larger area than those observed in eGFP-expressing HEK293 cells (Fig. 3d & g, red arrow). Interestingly, HEK293 cells with sodium arsenite treatment show NUP62 colocalization with G3BP1^+^ stress granules. However, these stress granules are absent of NUP54 (Supplementary Figure 4h). This is in contrast with both NUP62 and NUP54 colocalization to GR_50_^+^ RNA granules (Fig. 2g, Supplementary Figure 3a). Thus, nucleoporin colocalization with cytoplasmic granules is likely dependent on cellular stressor.

**Fig. 4:**
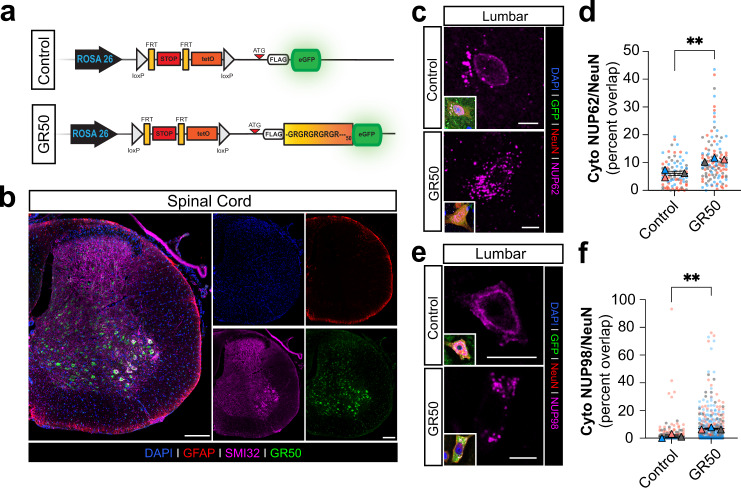
Poly-GR expression coincides with loss of perinuclear and increased cytosolic NUP62 in vivo. **a** Top: Schematic of Flexible Accelerated Stop Tetracycline Operator (F.A.S.T.) Cassette driven by the ROSA26 promoter at the ROSA26 locus used to express ATG-driven FLAG-GR50-GFP (GR50) in C57BL/6 mice. GR50 expression occurs upon crossing with a CAG-Cre mouse to excise the floxed STOP codon upstream of GR50. Bottom: The same cassette lacking the randomized GR50 sequence but still expressing eGFP is used as a control. **b** Representative images of GR50 (green) expression in the lumbar spinal cord of a GR50 mouse. GFAP (red) and SMI32 (magenta) denote astrocytes from neurons, respectively. Similar observations were made in a minimum of three biologically independent samples. Scale bar = 200 µm **c** Representative images of NUP62 (magenta) in GR50 (green) positive vs control in a 12-month-old mouse. Scale bar = 10 µm. **d** Quantification of NUP62 cytosolic puncta within NeuN-positive neurons of the lumbar spinal cord in 12-month-old mice. Value shown as percent of NUP62 fluorescence overlap within the whole cell ROI denoted by NeuN fluorescence. NeuN-positive cells (m) from *n* = 3 mice. Mean ± s.e.m. Unpaired t-test. **p = 0.0055. m = 90 control; 124 GR50. **e** Same as in (C) but for NUP98. **f** Same as in (D) but for NUP98. NeuN-positive cells (m) from *n* = 3 mice. Mean ± s.e.m. Unpaired t-test. **p = 0.0074. m = 276 control; 448 GR50.

To determine if GR_50_ mislocalization of NUP62 in vitro also occurs in vivo, we quantified NUP62 localization in a newly generated transgenic GR_50_ mouse model (Fig. 4a, b)^108^. A GR_50_-eGFP transgene was introduced by a Flexible Accelerated Stop Tetracycline Operator (F.A.S.T.) Cassette and driven by a ROSA26 promoter in C57BL/6 mice (Fig. 4a). Immunostaining for GR_50_-eGFP reveals its expression in tissue sections of lumbar spinal cord SMI32^+^ neurons (Fig. 4b). Analysis of NUP62 localization in the GR_50_-eGFP mouse model via immunostaining showed nuclear depletion and cytoplasmic accumulation of NUP62 protein (relative to eGFP control) in NeuN^+^ neurons of the lumbar spinal cord in 12-month-old adult animals (Fig. 4c). Total cytoplasmic NUP62 droplet surface area was significantly higher in GR_50_-eGFP mice than controls (Fig. 4d). Furthermore, GR was previously shown to affect localization of NUP98, another FG nup^61,95^. Thus, we conducted immunohistochemistry for NUP98 and found higher cytoplasmic levels in NeuN^+^ spinal neurons of GR_50_ mice (Fig. 4e & f). These in vivo data are consistent with our in vitro findings and indicate that cellular GR accumulation promotes the nuclear loss and cytoplasmic enrichment of FG nups including NUP62 and NUP98.

### Cytoplasmic NUP62:TDP-43 interactions promote insolubility

**Fig. 5:**
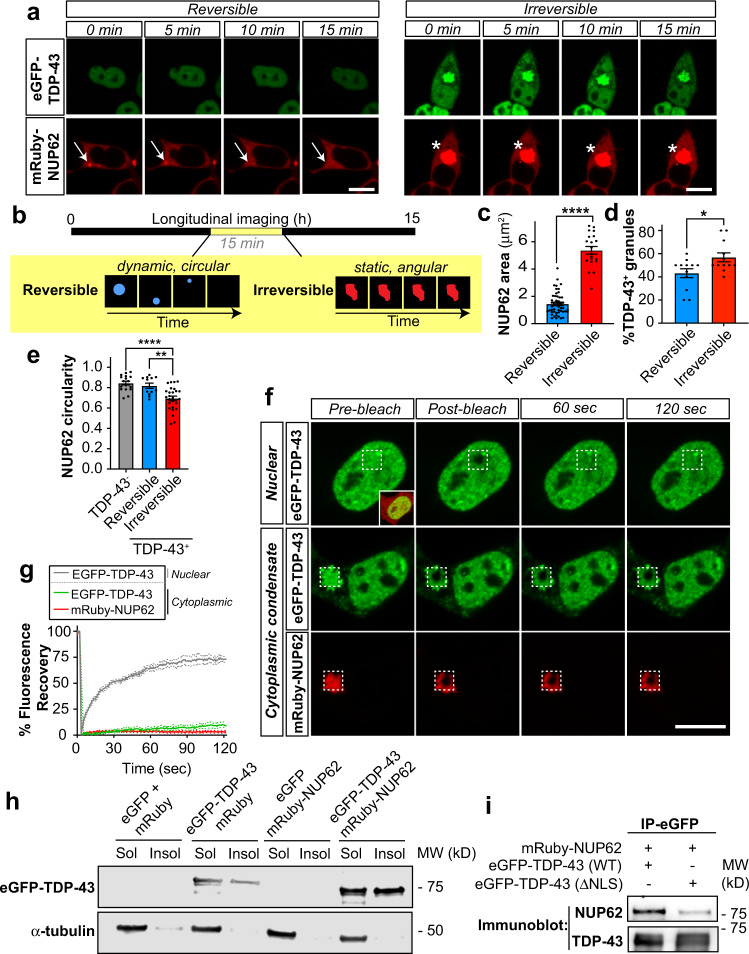
Cytoplasmic NUP62 and TDP-43 colocalization promotes insolubility. **a** HEK293 cells were co-transfected with mRuby-NUP62 and eGFP-TDP-43 (wild type). The cells were observed through live-scan confocal microscopy starting 3 h after transfection and images were obtained every 5 min over the course of 15 h. Two populations of cytoplasmic mRuby-NUP62 condensates were observed: reversible or irreversible. Reversible structures exhibit more dynamic activity and appear circular (see arrow). Irreversible structures appear less mobile or more static and have an angular structure (see asterisks). **b** Schematic depicting characteristics of cytoplasmic mRuby-NUP62 structures is shown at bottom. Representative still images were obtained from the 6–10 h time points of the imaging session. **c** Quantification of NUP62 area in confocal microscopy images obtained during live imaging (5–15 h timepoints) described in Fig. 5A, B. Irreversible condensates were significantly larger than reversible structures. The size of reversible granules was determined at times point immediately prior to dissipation. Irreversible granule area was calculated at final time point collected during live imaging session. *n* = 50 (reversible), 20 (irreversible) NUP62^+^ granules. Statistical significance was determined by two-tailed, unpaired student’s t-test. Data are shown as mean + /- SEM. **d** The percentage of reversible and irreversible mRuby-NUP62 granules containing eGFP-TDP-43 were calculated for each frame taken throughout the duration of living imaging session (5–15 h timepoints) described in Fig. 5a, b. A greater percentage of irreversible mRuby-NUP62 condensates contained eGFP-TDP43. *n* = 12 frames per group. Statistical significance was determined by two-tailed, unpaired student’s t-test. Data are shown as mean + /- SEM. **e** mRuby-NUP62 condensates were characterized for circularity score at the final time point of live image session (5–15 h timepoints) described in Fig. 5a, b. Irreversible mRuby-NUP62 + eGFP-TDP-43^+^ condensates (*n* = 26 condensates) had a significantly reduced circularity score relative to eGFP-TDP-43^-^ (*n* = 17 condensates) and reversible mRuby-NUP62 + eGFP-TDP-43^+^ condensates (*n* = 12 condensates). Statistically differences were calculated by one-way ANOVA with Tukey post hoc analysis. Data are shown as mean + /- SEM. **f** Representative FRAP analysis images of nuclear eGFP-TDP-43 (reference solubility control) and cytoplasmic eGFP-TDP-43 and mRuby-NUP62 condensates. **g** Quantification of FRAP analysis shows reduced fluorescence signal recovery in cytoplasmic eGFP-TDP-43 and mRuby-NUP62 condensates relative to nuclear eGFP-TDP-43 control. Data are shown as mean + /- SD. **h** HEK293 cells were transfected with indicated plasmids for 24 h. Soluble and insoluble biochemical fractionation was then conducted, and Western blot analysis was performed to evaluate TDP-43 and GAPDH (protein loading control). mRuby-NUP62 promotes the formation of increased insoluble TDP-43. Representative western blot image is shown. **i** HEK293 cells were transfected with mRuby-NUP62 and eGFP-TDP-43 (WT or ΔNLS) for 24 h. Samples were then immunoprecipitated by ChromoTek GFP-Trap Magnetic Agarose affinity beads. Samples were then immunoblotted for NUP62 and TDP-43. *p ≤ 0.05; **p ≤ 0.01; ****p ≤ 0.0001 vs control. Scale bar: 10 µm.

Given the colocalization of NUP62 and TDP-43 within cytoplasmic GR-induced RNA granules (Fig. 2), we next tested the consequence of NUP62:TDP-43 interactions on their protein dynamics. We generated a fluorescent mRuby-NUP62 expression plasmid that forms cytoplasmic NUP62 droplets when expressed in HEK293 cells. HEK293 cells were co-transfected with mRuby-NUP62 and eGFP-TDP-43 (wild type) and 6 h after transfection, cells were monitored by longitudinal imaging for 15 h. mRuby-NUP62 formed cytoplasmic condensates that often colocalized with eGFP-TDP-43 (Fig. 5a). Notably, this mislocalization and colocalization with TDP-43 is not generalizable to all FG nups since expression of NUP153-eGFP forms nuclear condensates and does not result in the formation of cytoplasmic mCherry-TDP43 droplets (Supplementary Figure 5a). mRuby-NUP62 signals that did not colocalize with cytoplasmic eGFP-TDP-43 exhibited characteristics of dynamic liquid-like droplets and were mobile, fused, and dissipated over the course of minutes (Fig. 5a, b, Supplementary Movie 1; “reversible”). However, when colocalized with eGFP-TDP-43, mRuby-NUP62 condensates were static and formed nonspherical structures that remained present throughout the duration of the 15 h imaging session (Fig. 5a, b, Supplementary Movie 2; “irreversible”). We then characterized the mRuby-NUP62 signal throughout the longitudinal imaging session and found the “irreversible” structures were larger (Fig. 5c), more frequently colocalized with eGFP-TDP-43 (Fig. 5d) and showed reduced circularity (Fig. 5e) compared to reversible mRuby-NUP62 condensates. These features suggest that cytoplasmic NUP62 forms liquid protein droplets that, when colocalized with cytoplasmic TDP-43, exhibit altered dynamics characteristic of gel-like or solid-state assemblies. Consistent with this, FRAP analysis of colocalized mRuby-NUP62:eGFP-TDP-43 cytoplasmic condensates revealed that cytoplasmic eGFP-TDP-43 (middle row) and mRuby-NUP62 (bottom row) signals do not recover after photobleaching (Fig. 5f, g, Supplementary Movie 3) indicative of insolubility. In contrast, nuclear eGFP-TDP-43 (Fig. 5f, g: top row, Supplementary Movie 4) recovers after photobleaching. To ensure this was not an artifact of interactions driven by the NUP62 reporter orientation, we repeated this experiment with a reversed NUP62-mRuby construct and observed similar results (Supplementary Figure 5b, c). Consistent with these findings, we performed soluble/insoluble fractionation and Western blot analyses of HEK293 cells co-expressing eGFP-TDP-43 and mRuby-NUP62 and found that this co-expression elevated insoluble eGFP-TDP-43 levels (Fig. 5h, Supplementary Figure 5d) indicating that cytoplasmic NUP62:TDP-43 interactions promote the formation of pathological insoluble TDP-43, likely through deleterious phase transitions^109^. We validated the mRuby-NUP62:eGFP-TDP-43 interaction by co-immunoprecipitation. HEK293 cells were co-transfected with mRuby-NUP62 and eGFP-TDP-43 (WT and ΔNLS). Isolated eGFP-TDP-43 protein complexes through immunoprecipitation and immunoblotting for NUP62 and TDP-43 showed both NUP62 and TDP-43 in the co-immunoprecipitation samples but with reduced NUP62 levels in eGFP-TDP-43 (ΔNLS) co-immunoprecipitation group (Fig. 5i). Furthermore, we found that HEK293 cells co-expressing mRuby-NUP62 and eGFP-TDP-43 (ΔNLS) reporters did not colocalize (Supplementary Figure 5e) suggesting the TDP-43 NLS may be required for this pathologic interaction with NUP62. Together, these data show that cytoplasmic NUP62 and TDP-43 interactions, driven by the TDP-43 NLS, promote the soluble to insoluble transition of these proteins in cellular models.

### Nup62 is a modifier of expanded G4C2 expression toxicity in *Drosophila*

**Fig. 6:**
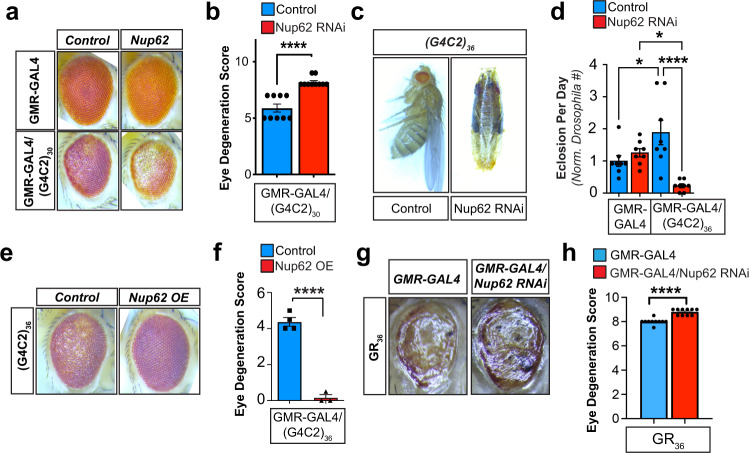
Nup62 is a genetic modifier of C9-ALS/FTD *Drosophila* models. **a** Representative images of fly eyes from GMR-GAL4 wild-type (top row) or (G4C2)_30_ repeat expansion (bottom row) flies combined with control (eGFP) or UAS-Nup62 RNAi flies. **b** Quantification of scored fly eye degeneration. Nup62 RNAi significantly enhances retinal degeneration of (G4C2)_30_ repeat expansion. Symbols are indicative of eye degeneration score for individual flies evaluated. *n* = 9 (control) or 10 (Nup62 RNAi) flies. Statistically significant differences were calculated by unpaired, two-tailed t-test: **** *p* ≤ 0.0001. Data are shown as mean + /- SEM. **c** Combination GMR-GAL4; (G4C2)_36_ repeat expansion were crossed with a UAS-Nup62 RNAi or UAS-eGFP fly. Shown are representative images of these flies 0–1 day post eclosion. **d** Bar graph shows the normalized quantification for (G4C2)_36_ repeat expansion fly eclosion in the presence and absence of Nup62 RNAi. The bars show average eclosion per day over the course of 8 days while individual dots are representative of fly counts for one 24 h eclosion period. (G4C2)_36_;GMR-GAL4 line crossed with UAS-eGFP fly line was used as the control group. Samples sizes *n* = 8 flies per group. Statistically significant differences in eclosion frequency were determined by one-way ANOVA with Tukey’s multiple comparison’s test: * *p* ≤ 0.05, **** *p* ≤ 0.0001. Data are shown as mean + /- SEM. **e** Combination GMR-GAL4/TM3; Nup62 OE/Sb were crossed with UAS-(G4C2)_36_ flies. Representative images are of flies carrying Nup62 overexpression or internal controls from the same cross carrying the Sb phenotype instead of Nup62 overexpression. Resulting progeny were evaluated within 24 h of eclosion. W1118 phenotype *Drosophila* were used as control. **f** Degeneration of fly eye was scored according to previously described methods^110^. We find Nup62 overexpression abolishes the (G4C2)_36_ repeat expansion fly eye degeneration (*n* = 4 flies per group). Statistically significant differences were calculated by unpaired, two-tailed t-test: **** *p* ≤ 0.0001. Data are shown as mean + /- SEM. **g** Representative images of retinal degeneration in the presence and absence of Nup62 knockdown with GR_36_ *Drosophila*. **h** Retinal degeneration was measured in GR_36_ *Drosophila* with and without Nup62 RNAi. Individual symbols are indicative of individual flies and the average degeneration score is presented by the bars. Sample size: *n* = 10 flies per group. Statistically significant differences were calculated by unpaired, two-tailed t-test. **** *p* ≤ 0.0001. Data are shown as mean + /- SEM.

Our observations indicate that NUP62 mislocalization is a feature of C9-ALS/FTLD models but the implications of NUP62 dysregulation on neuron survival is not known. To address this, we performed a genetic screen in the (G4C2)_30_-expressing *Drosophila* to determine whether FG nup depletion modulates neurotoxicity associated with the rough eye phenotype (Fig. 6a, b, Supplementary Figure 6a-c). The GMR-GAL4 system was used to drive (G4C2)_30_ and FG nup shRNA expression in the fly eye. Reduction of mammalian conserved FG nups through RNAi was validated through RT-qPCR (Supplementary Figure 6d-g) and progeny rough eye phenotype was scored in a blinded manner^110^. Expression of the (G4C2)_30_ repeat expansion induces the rough eye phenotype when compared to control (UAS-eGFP) *Drosophila* (Fig. 6a, Supplementary Figure 6b)^34^. Downregulation of specific FG nups that increased the rough eye phenotype degeneration score were classified as enhancers while those that mitigated the rough eye phenotype degeneration were suppressors (Supplementary Figure 6a). Nup62 knockdown significantly enhances (G4C2)_30_-mediated eye degeneration (Fig. 6a, b). However, (G4C2)_30_ flies with Nup98 RNAi do not exhibit altered rough eye phenotype and Nup58 and Nup153 loss suppresses the rough eye phenotype (Supplementary Figure 6b, c). Importantly, RNAi-mediated knockdown of FG nups alone does not alter eye phenotype in control (UAS-eGFP) *Drosophila* (Fig. 6a, Supplementary Figure 6b, top row). This suggests that any RNAi-mediated changes were specific to a genetic interaction with expression of the (G4C2)_30_ transgene. These data show that Nup62 abnormalities may contribute to disease pathogenesis, as Nup62 was a potent enhancer of eye neurodegeneration when its levels are reduced.

Our data indicate that GR promotes cytoplasmic NUP62 localization (Figs. 2–4). Notably, DPRs are detected at low levels and after degeneration in the (G4C2)_30_ repeat fly eye^34,60^. Therefore, to validate our initial screen and determine whether higher DPR levels enhance toxicity association with Nup62 downregulation, we also tested the impact of Nup62 knockdown on (G4C2)_36_ *Drosophila* retinal degeneration. This model was previously shown to produce an abundance of DPRs (GR and GP)^105^ and Nup62 loss in GMR-GAL4 (G4C2)_36_ *Drosophila* causes robust pupal lethality and eclosion defects (Fig. 6c, d, Supplementary Figure 6h). Together, these data confirm that a genetic interaction exists between Nup62 and repeat RNA but the elevated DPR burden exacerbates the resulting *Drosophila* phenotype. We generated a UAS-Nup62 expression *Drosophila* line (Nup62 OE) to test if Nup62 overexpression is sufficient to rescue the fly eye degeneration in these models (Supplementary Figure 6i)^109^. Nup62 OE *Drosophila* were crossed with the GMR-GAL4 (G4C2)_36_ repeat expansion fly and rough eye phenotypes were then scored as a measure of neurotoxicity in the adult progeny. Nup62 overexpression completely abolishes the rough eye phenotype in (G4C2)_36_-expressing *Drosophila* (Fig. 6e, f) and Nup62 overexpression modestly reduces GMR-GAL4/(G4C2)_30_ fly eye degeneration (Supplementary Figure 6j-k). To assess whether altering Nup62 levels modifies the GR-expressing *Drosophila* phenotype, we employed the GMR-GAL4xGR_36_ *Drosophila* which has a robust phenotype with reduced survival, significant retinal degeneration, extensive deterioration of eye size, and an absence of ommatidial organization (Fig. 6g)^105^. Nup62 RNAi mildly enhanced the GMR-GAL4xGR_36_ fly eye degeneration and reduced fly eclosion (Fig. 6g, h, Supplementary Figure 6l). Nup62 OE rescues GMR-GAL4xGR_36_ fly eye degeneration and we observed a mild reduction in GR_36_ eye deterioration (Supplementary Figure 6m). These data provide in vivo evidence that Nup62 modulates C9-ALS/FTLD neurotoxicity and is linked to G4C2 repeat expansion through GR accumulation.

### FG nups colocalize with phosphorylated ALS/FTLD TDP-43 pathology

**Fig. 7:**
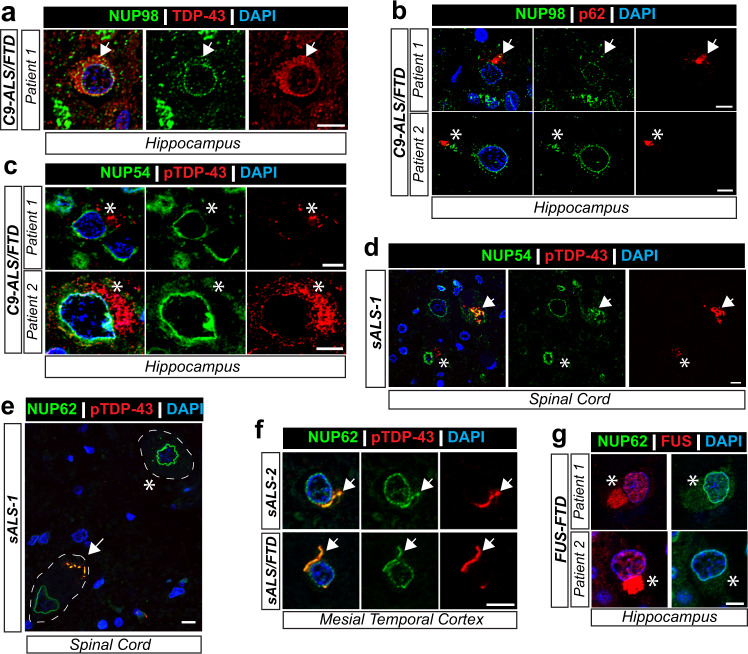
FG NUPs colocalize with TDP-43 proteinopathy in ALS/FTLD. NUP98, NUP54 or NUP62 (green) and total TDP-43, p62 or phosphoTDP-43 (pTDP-43, red) immunohistochemistry in postmortem tissue from various clinical diagnoses. **a** Cytoplasmic NUP98 is present in cells with cytoplasmic total TDP-43 (arrow) of C9-ALS/FTD postmortem hippocampal tissue. **b** Cytoplasmic NUP98 exhibits occasional colocalization (arrow) with p62^+^ inclusions in C9-ALS/FTD postmortem hippocampal tissue. However, cytoplasmic NUP98 was also found absent from p62^+^ inclusions (asterisks) in C9-ALS/FTD hippocampal tissue. **c** Phosphorylated TDP-43 was absent of cytoplasmic NUP54 in C9-ALS/FTD postmortem hippocampal tissue. **d** Postmortem sALS patient spinal cord tissue shows pTDP-43 inclusions that are both positive (arrow) and negative (asterisks) for NUP54. **e**, **f** Intact nuclear NUP62 signal surrounds DAPI^+^ nuclei (asterisks) but becomes more diffuse in cells exhibiting cytoplasmic pTDP-43 (arrow). Colocalization of NUP62 and pTDP-43 accumulations were observed in the spinal cord (E) and mesial temporal cortex (F) of sporadic ALS patient tissue. **g** Cytoplasmic FUS accumulations (red, asterisks) in the hippocampus from two FTLD patients do not show NUP62 localization. Scale bar: 10 µm.

pTDP-43 and NUP62 are colocalized in C9-ALS/FTD postmortem CNS tissue (Fig. 1). Similar to NUP62, several nucleoporins, like NUP98 and NUP54, contain low complexity domains and were shown to be disrupted by poly-GR and TDP-43^61,65,93,95^. Furthermore, we show that NUP54 and NUP98 colocalize with and are disrupted by GR_50_ expression in vitro (Supplementary Figure 3). Thus, we tested whether other FG nups colocalize with phosphorylated TDP-43 neuropathology in C9-ALS/FTD CNS tissue. Our NUP98 and pTDP-43 antibodies share the same species, so we used cytoplasmic total TDP-43 or p62 inclusions to assess colocalization with NUP98 by immunohistochemistry. We observed some, but limited, colocalization of cytoplasmic NUP98 with cytoplasmic TDP-43^+^ (Fig. 7a) and p62^+^ (Fig. 7b, Supplementary Table 6) inclusions in C9-ALS/FTLD dentate gyrus. Because p62 can label both pTDP-43^+^ inclusion or DPRs^111,112^, it is not clear whether this cytoplasmic NUP98 signal correlates with one or both of these neuropathological features. Since NUP54 colocalizes with GR_50_-induced TDP-43^+^ RNA granules in vitro, we evaluated if NUP54-pTDP-43 colocalization occurs in C9-ALS/FTLD and sporadic ALS (sALS) patient CNS tissue. We found NUP54 and pTDP-43 colocalization is not prevalent in C9-ALS/FTD hippocampal tissue (Fig. 7c, Supplementary Table 6). In contrast, NUP54 did colocalize with larger pTDP-43 inclusions (arrow) but not smaller pTDP-43 inclusions (asterisks) in sALS spinal cord samples (Fig. 7d). Notably, NUP54, NUP62, and NUP98 antibodies were also tested in control tissue and exhibited a uniform nuclear signal similar to the uniform nuclear nucleoporin signals observed in ALS/FTLD patient tissue without mislocalized or phosphorylated TDP-43 (Supplementary Figure 7a).

We next tested if NUP62 colocalized with TDP-43 proteinopathy in sporadic ALS/FTLD postmortem spinal cord and cortex (Fig. 7e, f, Supplementary Table 6). We observed a clear and uniform nuclear NUP62 signal around DAPI signal in cells without phosphorylated TDP-43 inclusions in ALS patient spinal cord (Fig. 7e, asterisk). In contrast, cells with cytoplasmic and phosphorylated TDP-43 deposits exhibit a disrupted nuclear NUP62 staining (Fig. 7e), consistent with earlier findings in C9-ALS/FTD postmortem tissue (Fig. 1). Interestingly, NUP62 colocalized with pTDP-43^+^ inclusions in the spinal cord and cortex of sALS/FTLD patients (Fig. 7e, f, arrowhead). These findings indicate that cytoplasmic NUP62 abnormalities occur in multiple ALS subtypes and are likely associated with aberrant TDP-43 inclusion formation.

To evaluate potential mechanisms underlying this co-pathology, we tested whether NUP62 abnormalities also occur in in vitro and in vivo models of aberrant TDP-43 liquid-liquid phase transitions and aggregation. We investigated NUP62 using in vitro and in vivo light-induced opto-TDP-43 models^98,113^ (Supplementary Figure 7). TDP-43 aggregation was induced in HEK293 cells transfected with optoTDP-43 constructs (Supplementary Figure 7b) followed by immunocytochemistry and super-resolution microscopy for NUP62. A blinded observer quantified nuclear NUP62 integrity using images and a scoring system (Supplementary Figure 7c-d, Supplementary Table 4) and nuclear NUP62 signal intensity (Supplementary Figure 7c, e-g) which showed nuclear NUP62 deficits associated with TDP-43 aggregation formation. Furthermore, NUP62 colocalized with cytoplasmic optoTDP43 inclusions (Supplementary Figure 7h). These findings were then validated in an optoTDP43 *Drosophila* model by performing immunostaining for FG nups by MAb414 antibody detection. We observed that FG nups colocalized to optoTDP43 inclusions in optoTDP43 *Drosophila* ventral nerve neurons that exhibited similar staining pattern as seen in ALS/FTLD patient tissue with phosphorylated TDP-43 inclusions (Supplementary Figure 7l).

FUS, another RNA binding protein, also forms pathological inclusions in a fraction of familial ALS and sporadic FTLD patients^4,5,8^. NUP62 abnormalities were recently associated with mutant FUS-linked ALS^114^. Therefore, we tested whether cytoplasmic accumulation of NUP62 also occurs in FUS-FTD postmortem tissue. Immunostaining of hippocampal samples from two separate FUS-FTD patients did not show detectable NUP62-FUS colocalization (Fig. 7g, Supplementary Table 6). Taken together, abnormal NUP62 localization can occur in sporadic ALS/FTLD associated with TDP-43 pathology but not in sporadic FUS-FTD.

## Discussion

Previous reports suggest C9-ALS/FTLD pathogenesis is primarily due to gain-of-function mechanisms. One consequence of toxic RNA and/or DPR accumulation is impaired nuc/cyto transport^39,60–63,115^ and abnormal NPC composition^88^ in C9-ALS/FTLD. FG nups comprise the selectivity barrier of the NPC and previous reports show perturbed nuclear FG nup staining in C9-ALS/FTLD, SOD1 ALS, and sporadic ALS/FTLD^88,116–118^. Evidence also shows FG nups colocalized with cytoplasmic TDP-43 accumulation^93,95^. However, the link between C9-ALS/FTLD pathobiology, nucleoporin irregularities, and TDP-43 proteinopathy is not fully defined. Here, we describe a mechanism in C9-ALS/FTLD in which the DPR GR induces NUP62 (and other FG nups) cytoplasmic mislocalization into TDP-43^+^ RNA granules. NUP62 and TDP-43 condensation and interaction result in the formation of insoluble TDP-43:NUP62 inclusions that are frequently observed C9-ALS/FTLD brain and spinal cord. We further show that NUP62 is present within TDP-43 inclusions in various in vitro and in vivo TDP-43 ALS/FTLD model systems not reliant on the C9orf72 repeat expansion such as the optoTDP-43 models^98,113^. Additionally, NUP62 is found within phosphorylated TDP-43 deposits in sporadic ALS/FTLD postmortem tissue. Taken together, this study outlines a mechanism in C9-ALS/FTLD where GR promotes the formation of TDP-43 inclusions alongside FG nup mislocalization and highlights a pathogenic process through which FG nup mislocalization could potentially promote TDP-43 proteinopathy in ALS/FTLD.

Previous work implicated nucleoporin mislocalization with TDP-43 overexpression^93^. Furthermore, FUS fibrils can drive TDP-43 phase separation and this was associated with nucleoporin:TDP-43 interactions in vitro^97^. This study investigates the pathogenic mechanisms underlying nucleoporin mislocalization and relationship to TDP-43 pathology in C9-ALS/FTLD. We discovered a robust colocalization of NUP62 with cytoplasmic pTDP-43 inclusions in C9-ALS/FTLD patient spinal cord and hippocampus (Fig. 1a, b). Furthermore, Pearson’s correlation analysis in C9-ALS/FTD iPSC neurons show a direct relationship between TDP-43 mislocalization and abnormal nuc/cyto NUP62 distribution (Fig. 1c, d). This supports the hypothesis that cytoplasmic mislocalization of NUP62 is associated with aberrant TDP-43 mislocalization and inclusion formation. Recent work similarly showed that GFP-(GR)_200_ promotes the cytoplasmic mislocalization of a transiently expressed TDP-43-Myc protein in vitro and that (GR)_20_ is sufficient to the drive aberrant phase separation and aggregation of purified TDP-43 in vitro^95^. We found that cytoplasmic GR accumulation through overexpression induces the formation of RNA granules that recruit endogenous TDP-43 and SG proteins (Fig. 2a, b)^119–121^. This is supported by studies that showed GR initiates the spontaneous formation of SGs with abnormal dynamics^54,122^. In contrast to our study, the previous study did not observe cellular colocalization of GR with G3BP1 or TDP-43 proteins but did identify direct interactions between GR:G3BP1 through co-IP analyses^54^. Furthermore, cytoplasmic GR:TDP-43 granules colocalize with endogenous NUP62 (Fig. 2g, h, Supplementary Figure 2). Thus, we hypothesize that GR:G3BP1:TDP-43:NUP62 assemblies are pathogenic since previous work indicated that GR perturbs SG dynamics (likely though aberrant liquid-liquid phase separation), which is thought to promote TDP-43 deposition^54,56^.

Consistent with our in vitro findings, previous studies showed that poly-GR colocalizes with cytoplasmic pTDP-43 inclusions in patient tissue^94^. AAV mediated GFP-(GR)_200_ expression in rodents is also sufficient to induce the formation of cytoplasmic structures that colocalized with some nucleoporins, SG markers, and TDP-43 proteins in vivo^95^. Notably, while the AAV GFP-(GR)_200_ model does show neurodegeneration, the transgenic GR_50_ mouse used in these studies does not^123^. We hypothesize this is due to the absence of a cytoplasmic TDP-43 burden in the GR_50_ mouse model. However, we do observe cytoplasmic NUP62 abnormalities in the transgenic GR_50_ mouse model which suggests that TDP-43:NUP62 condensates are a downstream event preceded by GR:NUP62 condensates. This is further supported by our observation that GR_50_ expression in HEK293 cells does not cause TDP-43 aggregation despite abnormal cytoplasmic NUP62 localization (Fig. 3 & 4) and TDP-43 condensates only form in response to very high accumulations of cellular GR (Supplementary Figure 2). Thus, GR_50_ likely initiates NUP62 mislocalization through sequestration to GR-induced RNA granules which, in turn, recruit TDP-43 that evolve into pathological inclusions.

One hypothesized pathway driving deleterious TDP-43 phase transitions and subsequent aggregation is through aberrant RNA granule dynamics which promote TDP-43 hyperphosphorylation^96^. In contrast to the GR-induced SGs observed in these studies, NUP62 does not localize to sorbitol- and sodium arsenite-induced SGs^124^. However, recent work in which FUS fibrils were used to induce liquid demixing of cytoplasmic TDP-43 did reveal some colocalization with NUP62 over time^97^ raising the possibility that the NUP62:TDP-43 interaction is pathogenic in nature. Supportive of this notion, we observed that cytoplasmic NUP62 interacts with and alters the dynamics and solubility of a TDP-43 reporter protein (Fig. 5f-i and Supplementary Figure 5b-d). Thus, we propose a process through which cytoplasmic NUP62 mislocalization and subsequent interaction with TDP-43 promotes TDP-43 insolubility, highlighting a pathobiology potentially resulting from FG nup mislocalization in ALS/FTLD.

TDP-43 inclusions are a neuropathological feature of several neurodegenerative diseases; thus NUP62’s role in promoting TDP-43 insolubility may be broadly relevant to a variety of neurodegenerative disorders. This is supported by early work in this study that indicates NUP62 is recruited to inclusions in both in vitro and in vivo models of TDP-43 aggregation (Supplementary Figure 7). Furthermore, early NUP62:TDP-43 assemblies may act through a feed-forward mechanism to promote TDP-43 nuclear loss-of-function through a seeding event or cytoplasmic gain-of-function toxicity via inclusions formation. Consistent with this, abnormal nuc/cyto transport was shown to occur independent of TDP-43 mislocalization in C9-ALS/FTLD iPSC neurons with normal nuclear NUP62 integrity^88^. Thus, while other pathways likely initiate transport defects observed in C9-ALS/FTLD, this is likely exacerbated by cytoplasmic TDP-43 accumulation and NUP62:TDP-43 colocalization in late-stage disease.

Collectively, this study defines C9-ALS/FTLD pathobiology wherein poly-GR accumulation drives the cytoplasmic mislocalization of NUP62 and  TDP-43 and their  interaction promotes a liquid-to-solid transition. We hypothesize that NUP62:TDP-43 interactions are not exclusive to C9-ALS/FTLD since NUP62 colocalizes  with pTDP-43 inclusions in sporadic ALS and FTLD postmortem tissue. This suggests that NUP62 mislocalization may be a common pathogenic event that drives TDP-43 proteinopathy across a variety of neurodegenerative disorders.

## Methods

### Ethical Approval

Experiments were conducted in compliance with ethical regulations approved by Institutional Biosafety Committee at the University of Pittsburgh. All procedures involving mice were approved by the Institutional Animal Care and Use Committee at Thomas Jefferson University.

### Immunohistochemistry staining and human tissue analysis

Deidentified human tissue from control, sporadic and C9orf72 ALS/FTD cases was obtained from the University of Pittsburgh Neuropathology Department Brain Bank and cases are described in Supplementary Table 1. Tissue collection and all experimental studies were reviewed and approved by the Committee for Oversight of Research and Clinical Training Involving Decedents (CORID) at the University of Pittsburgh. Paraffin embedded tissue sections from the cervical spinal cord, hippocampus, and mesial temporal cortex were stained with NUP62 (BD Biosciences, 610497, 1:100), NUP54 (Sigma-Aldrich, Cat. No. HPA-035929, 1:200), NUP98 (Abcam, Cat. No. ab50610, 1:250), phospho-TDP43 1D3 (Millipore Sigma, Cat. No. MABN14 or Biolegend, Cat. No. 829901, 1:200), TDP-43 (Proteintech, 10782-2-AP, 1:100), p62 (BD Transduction Laboratory, 610833, 1:200) and/or FUS (Sigma Aldrich, Cat. No. HPA008784, 1:200) as previously described^98^. Fluorescent images were captured with 60x objective on Nikon A1R confocal microscope. NUP62 secondary antibody was 488/FITC while phospho-TDP43 and FUS were labeled with 594/TRITC/Cy3 to ensure any localized signal detected was not due to bleed through between channels.

### Induced pluripotent stem cell maintenance and motor neuron differentiation

Induced pluripotent stem cell (iPSC) lines studied are described in Supplementary Table 2. iPSCs were maintained in mTeSR1 medium and cells exhibiting characteristics of spontaneous differentiation were removed prior to initiating differentiation protocol. Motor neuron differentiation was conducted as previously described^39,125^. Briefly, iPSC colonies were dissociated into a single cells suspension and plated at approximately 1,000,00 cells per well on a 6-well plate. iPSCs were then differentiated towards a motor neuron phenotype over the course of two stages. Both stages of iPSC differentiation consist of daily media changes and supplementation of N2B27 base media (50% DMEM F12, 50% Neurobasal, 1x NEAA, 1x Glutamax, 1x N2, 1x B27). The first stage of neuroectoderm induction (6 days) occurs once the plated iPSCs reach 90% confluency and upon confluency they are treated with N2B27 base media supplemented with 10 µM SB431542 and 100 nM LDN-193189. To mimic the signaling pattern of the ventral and posterior region of neural tube where motor neuron progenitors are located, media is also supplemented with 1 µM Retinoic Acid and 1 µM Smoothened Agonist. In the second stage (8 days) of neuronal differentiation, cultures are fed with N2B27 base media supplemented with 16 µM SU5402 and 10 µM DAPT. Patterning small molecules (Retinoic Acid and Smoothened Agonist) are also added to the media to assure the generation of spinal motor neurons. After the two-stage differentiation is completed, cells are dissociated and plated in NBM media (Neurobasal, 1x NEAA, 1x Glutamax, 1x N2, and 1x B27) supplemented with 0.2 µg/mL Ascorbic Acid, 10 ng/mL BDNF, 10 ng/mL GDNF, 10 ng/mL CNTF.

### Cell culture

HEK293 cells (ATCC) were maintained in DMEM (Thermo Fisher Scientific, Cat. No. 10313039) supplemented with 10% Hyclone Bovine Growth Serum (GE Healthcare Life Sciences, Cat. No. SH3054103HI), and 1x Glutamax (Thermo Scientific, Cat. No. 35050079) at 37°C, 5% CO_2_ regulated tissue culture incubator. Cells were plated on Collagen I Rat Protein, Tail (50 µg/mL, Thermo Fisher Scientific A1048301) coated glass for experiments requiring immunocytochemistry or plastic for Western blot lysates and cell death assays. Cells were transfected with 250 ng dipeptide repeat protein (50 repeat) and 150 ng optoTDP43 constructs at 80-90% confluency with Lipofectamine 3000 (Life Technologies, Cat. No. L3000001) for 24 h timepoints. Cell death was measured by Pierce LDH Cytotoxicity Assay kit according to the manufacturer’s protocol (Thermo Scientific, Cat. No. 88954). For optoTDP43 experiments: cells were transfected as described above and 4 h following transfection were exposed to blue-light (wavelength: 470 nm LED) in tissue culture incubator (37°C, 5% CO_2_) for 16–24 h. Dipeptide repeat protein expression plasmid DNA were kindly provided by Davide Trotti. For sodium arsenite-mediated stress granule induction, HEK293 cells were treated with 0.5 mM sodium arsenite for 45 minutes^98^.

### Immunocytochemistry

Cells were fixed in 4% paraformaldehyde. Following several 1x phosphate-buffered saline (PBS) washes, the cells were permeabilized in 0.3% TritonX-100 in 1x PBS before blocking in 5% normal donkey serum in 1x PBS with 0.3% TritonX-100 and incubated with primary antibody solution (10% normal donkey serum, 0.3% TritonX-100) overnight at 4°C. Another set of 1x PBS washes was then conducted prior to a 1 h secondary antibody (10% normal donkey serum, 0.3% TritonX-100) incubation. Coverslips were then washed with 1x PBS and mounted with Prolong Diamond with or without DAPI stain prior to microscopy imaging. Primary antibodies: MAb414 (1:1000, Biolegend, Cat. No. 902901), NUP62 (1:400, Abcam, Cat. No. ab188413 or 1:500, Millipore, MABE1043), MAP2 (1:1000, Synaptic Systems, Cat. No. 188044), NUP153 (1:300, Abcam, Cat. No. ab84872), NUP54 (2 ug/mL, Abcam, ab220890), NUP98 (1:500, Abcam, ab50610), TDP-43 (1:200, Proteintech, Cat. No.12892-1-AP or 10782-2-AP), ATAXIN-2 (1:400, Proteintech, Cat. No. 21776-1-AP), G3BP1 (1:300, Santa Cruz, Cat. No. sc-365338). Secondary antibodies: Donkey anti-Mouse (1:1000, Jackson Immunoresearch), Donkey anti-Rat (1:1000, Jackson Immunoresearch), Donkey anti-Guinea Pig (1:1000, Jackson Immunoresearch), Donkey anti-Rabbit (1:1000, Jackson Immunoresearch). Frequency of cytoplasmic NUP62 puncta were determined by spot detection for molecule with diameter of 1 µm or larger for 39-47 cells over the course of two separate experiments.

SYTO RNASelect Green Fluorescent stain (Thermo Fisher Scientific, Cat. No. S32703, 500 nM) was employed to determine whether RNA is present in GR_50_ condensates. Briefly, 24 h after transfection with GR_50_ plasmid DNA, HEK293 cells were fixed with ice-cold methanol for 20 min. Cells were then washed with 1x PBS before further immunofluorescent staining as described above.

### GR_50_ mouse studies

Characterization of NUP62 and NUP98 A knock-in F.A.S.T. cassette^126^ at the ROSA26 locus^127,128^ under the ROSA26 promoter^128,129^ was used for successful integration of FLAG-GR_50_-GFP or FLAG-GFP. GR_50_ was encoded using a randomized codon sequence to allow for production of the protein product absent of repeat-rich RNA. Animals were generated at Ingenious Targeting Laboratory, with successful knock-in confirmed via PCR from tail DNA samples. To elicit GR_50_/GFP expression, mice were crossed with CAG-Cre to allow for excision of STOP codon. CAG-Cre mice were a generous gift from Dr. Yuichi Obata, Riken BioResource Center, Japan. All mice are on a C57BL/6 background.

Twelve-month old, male mice were anesthetized and perfused by transcardial puncture with PBS and chilled 4% paraformaldehyde (PFA). Spinal cord was immediately dissected and post-fixed in 4% PFA for 24 hours, phosphate buffer for 24 h, and then 30% sucrose solution for a minimum of 48 hours until proper cryoprotection was ensured (ie. sample no longer floated). Spinal cord was embedded in O.C.T. Compound (Sakura, 4583) embedding medium. Samples were sectioned serially in the transverse orientation at a thickness of 30 µm and collected on glass slides. Slides were stored at -20°C until analysis.

For NUP62, frozen sections were processed for immunofluorescent confocal microscopy as previously described^130^ with modifications. Briefly, spinal cord sections were rinsed with PBS prior to a 20-min permeabilization with 0.5% T-X100 in HMK buffer (20 mM Hepes, pH 7.5, 1 mM MgCl_2_, 100 mM KCl). Sections were blocked in 10% normal donkey serum, 1% BSA in HMK buffer for 30 minutes. Sections were incubated with the following primary antibodies for approximately 18 hours at 4°C with gentle rotating: anti-NUP62 (BD Biosciences 610497, ms 1:400); anti-GFP (Millipore Sigma AB16901, chk 1:2500); and anti-NeuN (Cell Signaling Technology 24307, rb 1:200). Following 3x rinse in HMK buffer, sections were incubated with the following secondary antibodies at 1:500 each: Alexa Fluor 647 goat-anti-mouse (Thermo Fisher A32728), Alexa Fluor 546 donkey-anti-rabbit (Thermo Fisher A10040), and Alexa Fluor 488 goat-anti-chicken (Thermo Fisher A11039) for 1.5 h in HMK buffer with 1% BSA. Sections were rinsed 4x for 10 minutes each in HMK buffer at room temperature with gentle rotating, followed by 1x rinse in water and mounted in VectaShield mounting medium with DAPI (Vector).

For NUP98, frozen sections were processed for immunofluorescent confocal microscopy as previously described^95^ with modifications. Slides were steamed for 30 min in Cis-tris buffer (pH 6.0), followed by a 20-min wash in distilled H_2_O. Sides were rinsed 3x, 5 mins each with TBST. Slides were then blocked with DAKO all-purpose blocker (Agilent Technologies, Cat. No. X090930-2) for 1 hour at room temperature. Slides were incubated overnight with the following antibodies diluted in DAKO antibody diluent (Agilent Technologies, Cat. No. S302281-2): anti-NUP98 (abcam, rt 1:200); anti-GFP (Millipore Sigma AB16901, chk 1:2500); and anti-NeuN (Cell Signaling Technology 24307, rb 1:200). Slides were rinsed with TBST 3x, 10 minutes each and incubated for 2 hours with the following secondaries at 1:500 each: Alexa Fluor 546 goat-anti-rat (Thermo Fisher A11081), Alexa Fluor 647 donkey-anti-rabbit (Thermo Fisher A31573), and Alexa Fluor 488 goat-anti-chicken (Thermo Fisher A11039). Slides were rinsed 3x with TBST, followed by 1x rinse in water and mounted in VectaShield mounting medium with DAPI (Vector).

Imaging and quantification were done on a Nikon A1R-SI confocal microscope using NIS Elements software. Statistical analysis was performed using Graph Pad software.

### Drosophila studies

#### Drosophila stocks

Fly stocks and crosses were maintained on standard cornmeal medium in light/dark controlled incubators. RNAi flies were obtained from VDRC or the Transgenic RNAi project^131^ via Bloomington DGRC. The Nup62 overexpression fly was generated by the BestGene Inc methodology we used previously^113,132,133^. UAS-(G4C2)_30_ was kindly shared with our lab by Peng Jin^34^ and UAS-(G4C2)_36_ and UAS-GR_36_ flies were generous gifts from Adrian Isaac’s lab^49^.

#### Nup RNAi screen

UAS-RNAi virgin females were crossed with recombinant GMR-GAL4/UAS-(G4C2)_30_ males or control GMR-GAL4/UAS-EGFP males at 28°C. Female progenies of the appropriate genotype were collected, and their eyes were imaged with a Leica M205C digital camera at 0-1 days post-eclosion. Images of external eye phenotype were then scored as previously described^110^. Briefly, fly eyes were objectively scored according to the presence of absence of retinal collapse, ommatidial array disorganization, ommatidial pitting, ommatidial fusion, abnormal bristle orientation and supernumerary IOB. A higher score in this system is indicative of greater eye degeneration.

#### Nup62 RNAi, G4C2 and poly-GR toxicity characterization

GMR-GAL4/UAS-(G4C2)_36_ males were crossed with Nup62 RNAi virgin females at 25°C and imaged/quantified. GMR-GAL4/UAS-(G4C2)_36_ males were crossed with Nup62 OE virgin females and external eyes were imaged and quantified as described above. Recombinant GMR-GAL4/UAS-Nup62 RNAi and GMR-GAL4/UAS-Nup62 overexpression lines were generated and crossed with previously described UAS-GR_36_^105^ or an UAS-EGFP control^55^ at 25°C. As described earlier, eyes were imaged within 24 h of eclosion. Pupal lethality and eclosion defects were quantified by counting the number of animals that emerged from their pupal case every 24 h time-period for 8 days and we used 2 females and 2 males for the eclosion assay (3-5 replicates). Genetic modifier screens are depicted in schematic that is shown in Supplementary Figure 6a.

#### Optogenetic-TDP43 Drosophila

Blue-light studies were conducted as previously described^113^. Briefly, UAS-optoTDP43 females were crossed with OK371-GAL4 males in the dark for 120 h to assess the impact of OptoTDP43 on Drosophila motor neurons. Third instar larvae were then collected and exposed to dark or 14 watt 465 nm blue LED light for 24 h. Larval preparations were then prepared as previously described^134^. Briefly, larvae were dissected in ice-cold PBS and then fixed in 4% formaldehyde. Following a series of PBS washes and 5% TritonX-100 permeabilization, samples then underwent MAb414 (1:1000, Abcam, ab24609) immunofluorescent labelling. Images were then collected by confocal microscopy to determine whether FG nucleoporins are altered in this model system.

### Biochemical fractionation

Detergent solubility assay was performed as previously described but with minor modifications^98^. Briefly, HEK293 cells were transfected with plasmid DNA for 24 h. Cells were then washed with 1x PBS. Samples were then collected on ice following a 10 min incubation in RIPA buffer (25 mM Tris-HCL pH 7.6 (Sigma-Aldrich), 150 mM NaCl (Millipore), 5 mM EDTA (Sigma Aldrich), 1% TritonX-100 (Sigma-Aldrich), 1% sodium deoxycholate (Sigma Aldrich), 0.1% SDS (Fisher Scientific)), protease inhibitor cocktail (Sigma Aldrich), and 1 mM PMSF (Thermo Fisher Scientific) before being sonicated (10 × 3 s pulses on ice). Samples were then centrifuged at 16,000x *g* for 30 min at 4°C. The supernatant (detergent-soluble fraction) was then isolated and saved until further characterization.

### Immunoprecipitation

Cell lysates were collected in cell lysis buffer (Cat. No. 9803, Cell Signaling Technologies) supplemented with protease inhibitor cocktail (Cat. No. P8340, Sigma Aldrich) for 30 min on ice and then centrifuged for 17,000x *g* for 10 min at 4°C. GFP-Trap Magnetic Agarose (Cat. No. gtma, chromotek) beads were equilibrated in dilution buffer (10 mM Tris/Cl pH 7.5, 150 mM NaCl, 0.5 mM EDTA) before adding to the cell lysates. The samples were then incubated with GFP-Trap Magnetic Agarose beads for 3 hours at 4°C and washed three times in wash buffer (10 mM Tris/Cl pH 7.5, 150 mM NaCl, 0.5 mM EDTA). Samples were then eluted in 2x SDS-sample buffer (120 mM Tris/Cl pH 6.7, 20% glycerol, 4% SDS, 0.04% bromophenol blue, 10% β-mercaptoethanol). Prepared samples were boiled at 95°C for 5 minutes and then analyzed by immunoblot.

### Immunoblot

Samples were collected and prepared as described for biochemical fractionation or immunoprecipitation. Then, samples were separated by gel electrophoresis on 4-20% polyacrylamide gel (Bio-Rad, Cat. No. 4561094) and transferred to nitrocellulose membrane. The membranes were then blocked in 5% nonfat dry milk prepared in TBS with 0.1% Tween-20 or Odyssey block (LI-COR Biosciences, Cat. No. 927-50000) after a series of water and 1x TBS washes. After the blocking step, the membranes were incubated in primary antibody solution (5% nonfat dry milk with 0.1% Tween-20 or Odyssey Block) at 4°C overnight. The membranes were then washed in 1x TBS with 0.1% Tween-20 followed by horseradish peroxidase (HRP)-conjugated or fluorescently labelled secondary antibody (Jackson Immunoresearch) solution (1% nonfat dry milk with 0.1% Tween-20 or Odyssey block) incubation. Immunoblots with HRP-conjugate secondary antibodies were then developed with chemiluminescence (Western Lightning ECL Pro, Perkin Elmer) and detected on Amersham ImageQuant 800 (Cytiva Life Sciences). Immunoblots labelled with fluorescent secondary antibodies were detected on LI-COR Biosciences Odyssey Imager. Primary antibodies: TDP-43 (Proteintech, Cat. No. 10782-2-AP, 1:2000), alpha-tubulin (Sigma Aldrich, Cat. No. T5168, 1:1000), NUP62 (Millipore, MABE1043, 1:500). Secondary antibodies: Donkey anti-Rabbit 680 (LI-COR Biosciences, Cat. No. 926-68073, 1:1000), Donkey anti-Rabbit 800 (LI-COR Biosciences, Cat. No. 926-32213, 1:1000), Donkey anti-Rabbit HRP (Jackson Immunoresearch, Cat. No. 711-035-152, 1:10,000), Donkey anti-Rat HRP (Jackson Immunoresearch, Cat. No. 712-035-150, 1:10,000).

### Quantitative reverse-transcriptase polymerase chain reaction (qRT-PCR)

*FG Nup knockdown validation in Drosophila* Frozen fly heads (minimum of 9 flies/group) were homogenized with Trizol (Cat. No. 15596026, Ambion) within 24 h of eclosion. RNA was isolated into the upper aqueous phase with chloroform addition and then precipitated out by isopropanol. RNA was then pelleted by centrifugation, washed with 75% ethanol solution, and air-dried prior to being resuspended in nuclease-free water. cDNA was synthesized from equal volumes of RNA samples by iScript Select cDNA Synthesis Kit (Cat. No. 170-8897, BioRad).

In vitro *transcript characterization* RNA was extracted and purified from cellular samples with miRNeasy Mini Kit (Cat. No. 217004, Qiagen). cDNA was synthesized from equal volumes of RNA samples by iScript Select cDNA Synthesis Kit (Cat. No. 170-8897, BioRad).

Primers (10 µM working concentrations) (Supplementary Table 3) were generated by Integrated DNA Technologies. qPCR reactions were prepared in SsoAdvanced Universal SYBR Green Supermix (Cat. No. 1725272, BioRad) and were run on BioRad CFX96 Real-Time System in triplicate technical replicates. Results were determined through analysis of the comparative Ct values^135^.

### Microscopy

Image acquisition of fixed samples were acquired on a Nikon A1 laser-scanning confocal system with 40X and/or 60X oil immersion objectives (CFI Plan Fluor 40X Oil; CFI Plan Apo Lambda 60X Oil, Nikon) or Nikon N-SIM super-resolution Microscope with 60X oil immersion objectives (Plan Apo TIRF 60X Oil) and Hamamatsu C11440 Orca Flash 4.0 camera. 3D SIM images were reconstructed and then deconvolved prior to analysis. Image analysis was conducted in NIS-Elements AR Analysis 4.51.

#### Nuclear-cytoplasmic (Nuc/Cyto) distributions

Proteins were detected by immunofluorescent staining following 4% paraformaldehyde fixation. Confocal microscopy images were then collected and maximum intensity project images were used for analysis. Regions of interest (ROI) were drawn around the nuclear and cytoplasmic regions. The nuclear region was identified by DAPI signal and the cytoplasmic region was according to cytoskeletal marker or plasmid fluorescent reporter construct.

*Live-cell imaging* All live-cell imaging was performed on Nikon A1 laser scanning confocal microscope outfitted with Tokai HIT stage-top incubator while utilizing 40x oil immersion objective. Stage-top incubator was allowed to equilibrate to 37 °C and 5% CO_2_ for 10 min prior to imaging. HEK293 cells were transfected for 6 prior to imaging session. Images were acquired every 5 min for 15 h.

*Fluorescence Recovery After Photobleaching (FRAP) Imaging* FRAP studies were conduct as previously described^98^. Briefly, a 60x oil immersion objective on confocal microscope was used to monitor condensates. ROI was drawn over structure of interest and reference ROI was included in an adjacent, non-bleached cell. Four to five baseline structure images were obtained and then structure was bleached for 500 ms using 50% laser power (488 nm or 594 nm laser lines). Structures were observed for 120 s. Data represents the fluorescence signal recovery of 11–20 structures.

### Nuclear integrity scoring and analysis

Following NUP62 immunostaining and imaging by structured illumination microscopy (SIM), nuclear NUP62 integrity and continuity were measured. Nuclear NUP62 integrity was scored by a blinded, unbiased observer. Nuclei that exhibited a fragmented or irregular pattern were given a lower nuclear integrity score (described in Supplementary Table 4). Furthermore, using ImageJ analysis software, we linearized the nuclear NUP62 signal and representative examples are shown above graphs (Supplementary Figure 7). The profile plot was then used to measure NUP62 signal across the length (in pixels) of the select NUP62 staining. The profile plots were normalized to maximum signal intensity to account for any variability in staining intensity and normalized to the length of measured signal to account for nuclear size variability. The area under the curve (AUC) for these profile plots was then calculated and averaged for each group (*n* = 7–10 nuclei).

### Plasmids

eGFP-TDP-43 (WT & ΔNLS) and optoTDP43 constructs were previously generated by our lab^98^. Other plasmid constructs were generated by Gibson Assembly. NUP62 (Gift from Akiko Takedo) and mRuby (from Addgene Plasmid# 54614) fragments were PCR-amplified. The fragments were then inserted at NotI and NheI (mRuby-NUP62) or BamHI (NUP62-mRuby) restriction enzyme sites of the mRuby2-N1 base vector (Addgene Plasmid# 54614). FLAG-DPR_50_-eGFP and FLAG-DPR_50_-mCherry were gifts from Davide Trotti. Lentiviral GR_50_ was created by PCR amplifying FLAG-GR_50_-eGFP and inserting fragment into BsrGI and BsiWI restriction enzyme sites of lenti dCAS-VP64_Blast (Addgene Plasmid # 61425) base vector. Primers are provided in Supplementary Table 5. Newly generated plasmids were validated by Sanger Sequencing (Genewiz).

### Statistical Analysis

Experimental data was collected, and outliers determined by ROUT’s outlier test (Q = 1%). Following removal of outliers, data sets are shown as the mean and standard error of the mean. Statistically significant differences between experimental groups were calculated by GraphPad Prism software (Version 7) and deemed significant when *p* ≤ 0.05. Statistically significant differences were determined by unpaired Student’s t-test when comparing two variables or one-way ANOVA with Dunnett or Tukey’s multiple comparisons test when comparing multiple. Statistical analysis of nuclear NUP62 levels in control and C9orf72 ALS iPSC neurons was conducted by two-tailed Mann-Whitney test. Details of statistical analyses are also provided in the figure legends.

### Reporting summary

Further information on research design is available in the Nature Research Reporting Summary linked to this article.

## Supplementary information

Supplementary Information

Description of Additional Supplementary Files

Supplementary Movie 1

Supplementary Movie 2

Supplementary Movie 3

Supplementary Movie 4

Reporting Summary

## Data Availability

The data that supports the findings of this study are available from the corresponding author upon request. Source data are provided with this paper.

## Source data

Source Data

## References

[1] Ling S-C, Polymenidou M, Cleveland DW (2013). Converging mechanisms in ALS and FTD: disrupted RNA and protein homeostasis. Neuron.

[2] Lattante S, Ciura S, Rouleau GA, Kabashi E (2015). Defining the genetic connection linking amyotrophic lateral sclerosis (ALS) with frontotemporal dementia (FTD). Trends Genet.

[3] Dormann D, Haass C (2011). TDP-43 and FUS: a nuclear affair. Trends Neurosci..

[4] Neumann M (2006). Ubiquitinated TDP-43 in frontotemporal lobar degeneration and amyotrophic lateral sclerosis. Science.

[5] Neumann M (2009). A new subtype of frontotemporal lobar degeneration with FUS pathology. Brain.

[6] Urwin H (2010). FUS pathology defines the majority of tau- and TDP-43-negative frontotemporal lobar degeneration. Acta Neuropathol..

[7] Kwiatkowski TJ (2009). Mutations in the FUS/TLS gene on chromosome 16 cause familial amyotrophic lateral sclerosis. Science.

[8] Arai T (2006). TDP-43 is a component of ubiquitin-positive tau-negative inclusions in frontotemporal lobar degeneration and amyotrophic lateral sclerosis. Biochem. Biophys. Res. Commun..

[9] Vance C (2009). Mutations in FUS, an RNA processing protein, cause familial amyotrophic lateral sclerosis type 6. Science.

[10] DeJesus-Hernandez M (2011). Expanded GGGGCC hexanucleotide repeat in noncoding region of C9ORF72 causes chromosome 9p-linked FTD and ALS. Neuron.

[11] Nguyen HP, Van Broeckhoven C, van der Zee J (2018). ALS Genes in the Genomic Era and their Implications for FTD. Trends Genet.

[12] Renton AE (2011). A hexanucleotide repeat expansion in C9ORF72 is the cause of chromosome 9p21-linked ALS-FTD. Neuron.

[13] Rademakers R (2012). C9orf72 repeat expansions in patients with ALS and FTD. Lancet Neurol..

[14] Ciura S (2013). Loss of function of C9orf72 causes motor deficits in a zebrafish model of amyotrophic lateral sclerosis. Ann. Neurol..

[15] Donnelly CJ (2013). RNA toxicity from the ALS/FTD C9ORF72 expansion is mitigated by antisense intervention. Neuron.

[16] Almeida S (2013). Modeling key pathological features of frontotemporal dementia with C9ORF72 repeat expansion in iPSC-derived human neurons. Acta Neuropathol..

[17] Shi Y (2018). Haploinsufficiency leads to neurodegeneration in C9ORF72 ALS/FTD human induced motor neurons. Nat. Med..

[18] Belzil VV (2013). Reduced C9orf72 gene expression in c9FTD/ALS is caused by histone trimethylation, an epigenetic event detectable in blood. Acta Neuropathol..

[19] van Blitterswijk M (2015). Novel clinical associations with specific C9ORF72 transcripts in patients with repeat expansions in C9ORF72. Acta Neuropathol..

[20] Balendra R, Isaacs AM (2018). C9orf72-mediated ALS and FTD: multiple pathways to disease. Nat. Rev. Neurol..

[21] Koppers M (2015). C9orf72 ablation in mice does not cause motor neuron degeneration or motor deficits. Ann. Neurol..

[22] O’Rourke JG (2016). C9orf72 is required for proper macrophage and microglial function in mice. Science.

[23] Jiang J (2016). Gain of Toxicity from ALS/FTD-Linked Repeat Expansions in C9ORF72 Is Alleviated by Antisense Oligonucleotides Targeting GGGGCC-Containing RNAs. Neuron.

[24] Atanasio A (2016). C9orf72 ablation causes immune dysregulation characterized by leukocyte expansion, autoantibody production, and glomerulonephropathy in mice. Sci. Rep..

[25] Hautbergue GM (2017). SRSF1-dependent nuclear export inhibition of C9ORF72 repeat transcripts prevents neurodegeneration and associated motor deficits. Nat. Commun..

[26] Stopford MJ (2017). C9ORF72 hexanucleotide repeat exerts toxicity in a stable, inducible motor neuronal cell model, which is rescued by partial depletion of Pten. Hum. Mol. Genet.

[27] Batra R, Lee CW (2017). Mouse models of c9orf72 hexanucleotide repeat expansion in amyotrophic lateral sclerosis/ frontotemporal dementia. Front. Cell Neurosci..

[28] Chew J (2015). Neurodegeneration. C9ORF72 repeat expansions in mice cause TDP-43 pathology, neuronal loss, and behavioral deficits. Science.

[29] Liu Y (2016). C9orf72 BAC Mouse Model with Motor Deficits and Neurodegenerative Features of ALS/FTD. Neuron.

[30] O’Rourke JG (2015). C9orf72 BAC transgenic mice display typical pathologic features of ALS/FTD. Neuron.

[31] Zhu Q (2020). Reduced C9ORF72 function exacerbates gain of toxicity from ALS/FTD-causing repeat expansion in C9orf72. Nat. Neurosci..

[32] Shao Q (2019). C9orf72 deficiency promotes motor deficits of a C9ALS/FTD mouse model in a dose-dependent manner. Acta Neuropathol. Commun..

[33] Cooper-Knock J (2014). Sequestration of multiple RNA recognition motif-containing proteins by C9orf72 repeat expansions. Brain.

[34] Xu Z (2013). Expanded GGGGCC repeat RNA associated with amyotrophic lateral sclerosis and frontotemporal dementia causes neurodegeneration. Proc. Natl Acad. Sci. USA.

[35] Haeusler AR (2014). C9orf72 nucleotide repeat structures initiate molecular cascades of disease. Nature.

[36] Conlon, E. G. et al. The C9ORF72 GGGGCC expansion forms RNA G-quadruplex inclusions and sequesters hnRNP H to disrupt splicing in ALS brains. *Elife* **5**, e17820 (2016). 10.7554/eLife.17820 PMC5050020 27623008

[37] Mori K (2013). hnRNP A3 binds to GGGGCC repeats and is a constituent of p62-positive/TDP43-negative inclusions in the hippocampus of patients with C9orf72 mutations. Acta Neuropathol..

[38] Celona, B. et al. Suppression of C9orf72 RNA repeat-induced neurotoxicity by the ALS-associated RNA-binding protein Zfp106. *Elife* **6**, e19032 (2017). 10.7554/eLife.19032 PMC5283830 28072389

[39] Ortega JA (2020). Nucleocytoplasmic Proteomic Analysis Uncovers eRF1 and Nonsense-Mediated Decay as Modifiers of ALS/FTD C9orf72 Toxicity. Neuron.

[40] Freibaum BD, Taylor JP (2017). The Role of Dipeptide Repeats in C9ORF72-Related ALS-FTD. Front. Mol. Neurosci..

[41] Cleary JD, Ranum LP (2017). New developments in RAN translation: insights from multiple diseases. Curr. Opin. Genet. Dev..

[42] Zu T (2011). Non-ATG-initiated translation directed by microsatellite expansions. Proc. Natl Acad. Sci. USA.

[43] Mori K (2013). Bidirectional transcripts of the expanded C9orf72 hexanucleotide repeat are translated into aggregating dipeptide repeat proteins. Acta Neuropathol..

[44] Mori K (2013). The C9orf72 GGGGCC repeat is translated into aggregating dipeptide-repeat proteins in FTLD/ALS. Science.

[45] Ash PEA (2013). Unconventional translation of C9ORF72 GGGGCC expansion generates insoluble polypeptides specific to c9FTD/ALS. Neuron.

[46] Gendron TF (2013). Antisense transcripts of the expanded C9ORF72 hexanucleotide repeat form nuclear RNA foci and undergo repeat-associated non-ATG translation in c9FTD/ALS. Acta Neuropathol..

[47] Sareen D (2013). Targeting RNA foci in iPSC-derived motor neurons from ALS patients with a C9ORF72 repeat expansion. Sci. Transl. Med..

[48] Cooper-Knock J (2015). Antisense RNA foci in the motor neurons of C9ORF72-ALS patients are associated with TDP-43 proteinopathy. Acta Neuropathol..

[49] Mizielinska S (2013). C9orf72 frontotemporal lobar degeneration is characterised by frequent neuronal sense and antisense RNA foci. Acta Neuropathol..

[50] White MR (2019). C9orf72 poly(pr) dipeptide repeats disturb biomolecular phase separation and disrupt nucleolar function. Mol. Cell.

[51] Xu W, Xu J (2018). C9orf72 Dipeptide Repeats Cause Selective Neurodegeneration and Cell-Autonomous Excitotoxicity in Drosophila Glutamatergic Neurons. J. Neurosci..

[52] Starr A, Sattler R (2018). Synaptic dysfunction and altered excitability in C9ORF72 ALS/FTD. Brain Res.

[53] Boeynaems S (2017). Phase separation of c9orf72 dipeptide repeats perturbs stress granule dynamics. Mol. Cell.

[54] Lee K-H (2016). C9orf72 Dipeptide Repeats Impair the Assembly, Dynamics, and Function of Membrane-Less Organelles. Cell.

[55] Wen X (2014). Antisense proline-arginine RAN dipeptides linked to C9ORF72-ALS/FTD form toxic nuclear aggregates that initiate in vitro and in vivo neuronal death. Neuron.

[56] Zhang Y-J (2018). Poly(GR) impairs protein translation and stress granule dynamics in C9orf72-associated frontotemporal dementia and amyotrophic lateral sclerosis. Nat. Med..

[57] Lopez-Gonzalez R (2016). Poly(GR) in C9ORF72-Related ALS/FTD Compromises Mitochondrial Function and Increases Oxidative Stress and DNA Damage in iPSC-Derived Motor. Neurons Neuron.

[58] Choi SY (2019). C9ORF72-ALS/FTD-associated poly(GR) binds Atp5a1 and compromises mitochondrial function in vivo. Nat. Neurosci..

[59] Hartmann H (2018). Proteomics and C9orf72 neuropathology identify ribosomes as poly-GR/PR interactors driving toxicity. Life Sci. Alliance.

[60] Zhang K (2015). The C9orf72 repeat expansion disrupts nucleocytoplasmic transport. Nature.

[61] Hayes, L. R., Duan, L., Bowen, K., Kalab, P. & Rothstein, J. D. C9orf72 arginine-rich dipeptide repeat proteins disrupt karyopherin-mediated nuclear import. *Elife* **9**, e51685 (2020). 10.7554/eLife.51685 PMC7051184 32119645

[62] Boeynaems S (2016). Drosophila screen connects nuclear transport genes to DPR pathology in c9ALS/FTD. Sci. Rep..

[63] Jovičić A (2015). Modifiers of C9orf72 dipeptide repeat toxicity connect nucleocytoplasmic transport defects to FTD/ALS. Nat. Neurosci..

[64] Freibaum BD (2015). GGGGCC repeat expansion in C9orf72 compromises nucleocytoplasmic transport. Nature.

[65] Shi KY (2017). Toxic PRn poly-dipeptides encoded by the C9orf72 repeat expansion block nuclear import and export. Proc. Natl Acad. Sci. USA.

[66] Hutten S (2020). Nuclear Import Receptors Directly Bind to Arginine-Rich Dipeptide Repeat Proteins and Suppress Their Pathological Interactions. Cell Rep..

[67] Görlich D, Mattaj IW (1996). Nucleocytoplasmic transport. Science.

[68] Cronshaw JM, Krutchinsky AN, Zhang W, Chait BT, Matunis MJ (2002). Proteomic analysis of the mammalian nuclear pore complex. J. Cell Biol..

[69] Patel SS, Belmont BJ, Sante JM, Rexach MF (2007). Natively unfolded nucleoporins gate protein diffusion across the nuclear pore complex. Cell.

[70] Ribbeck K, Görlich D (2002). The permeability barrier of nuclear pore complexes appears to operate via hydrophobic exclusion. EMBO J..

[71] Ribbeck K, Görlich D (2001). Kinetic analysis of translocation through nuclear pore complexes. EMBO J..

[72] Mohr D, Frey S, Fischer T, Güttler T, Görlich D (2009). Characterisation of the passive permeability barrier of nuclear pore complexes. EMBO J..

[73] Bischoff FR, Krebber H, Kempf T, Hermes I, Ponstingl H (1995). Human RanGTPase-activating protein RanGAP1 is a homologue of yeast Rna1p involved in mRNA processing and transport. Proc. Natl Acad. Sci. USA.

[74] Kusano A, Staber C, Ganetzky B (2001). Nuclear mislocalization of enzymatically active RanGAP causes segregation distortion in Drosophila. Dev. Cell.

[75] Bischoff FR, Klebe C, Kretschmer J, Wittinghofer A, Ponstingl H (1994). RanGAP1 induces GTPase activity of nuclear Ras-related Ran. Proc. Natl Acad. Sci. USA.

[76] Steggerda SM, Paschal BM (2002). Regulation of nuclear import and export by the GTPase Ran. Int Rev. Cytol..

[77] Macara IG (2001). Transport into and out of the Nucleus. Microbiol. Mol. Biol. Rev..

[78] Wente SR, Rout MP (2010). The nuclear pore complex and nuclear transport. Cold Spring Harb. Perspect. Biol..

[79] Aramburu IV, Lemke EA (2017). Floppy but not sloppy: Interaction mechanism of FG-nucleoporins and nuclear transport receptors. Semin. Cell Dev. Biol..

[80] Kapinos LE, Huang B, Rencurel C, Lim RYH (2017). Karyopherins regulate nuclear pore complex barrier and transport function. J. Cell Biol..

[81] Allen NP, Huang L, Burlingame A, Rexach M (2001). Proteomic analysis of nucleoporin interacting proteins. J. Biol. Chem..

[82] Rexach M, Blobel G (1995). Protein import into nuclei: association and dissociation reactions involving transport substrate, transport factors, and nucleoporins. Cell.

[83] Kim SJ (2018). Integrative structure and functional anatomy of a nuclear pore complex. Nature.

[84] Frey S, Görlich D (2007). A saturated FG-repeat hydrogel can reproduce the permeability properties of nuclear pore complexes. Cell.

[85] Denning DP, Patel SS, Uversky V, Fink AL, Rexach M (2003). Disorder in the nuclear pore complex: the FG repeat regions of nucleoporins are natively unfolded. Proc. Natl Acad. Sci. USA.

[86] Labokha AA (2013). Systematic analysis of barrier-forming FG hydrogels from Xenopus nuclear pore complexes. EMBO J..

[87] Solomon DA (2018). A feedback loop between dipeptide-repeat protein, TDP-43 and karyopherin-α mediates C9orf72-related neurodegeneration. Brain.

[88] Coyne, A. N. et al. G4C2 Repeat RNA Initiates a POM121-Mediated Reduction in Specific Nucleoporins in C9orf72 ALS/FTD. *Neuron* **107**, 1124–1140.e11 (2020). 10.1016/j.neuron.2020.06.027 PMC8077944 32673563

[89] Dewangan PS, Sonawane PJ, Chouksey AR, Chauhan R (2017). The Nup62 Coiled-Coil Motif Provides Plasticity for Triple-Helix Bundle Formation. Biochemistry.

[90] Chug H, Trakhanov S, Hülsmann BB, Pleiner T, Görlich D (2015). Crystal structure of the metazoan Nup62•Nup58•Nup54 nucleoporin complex. Science.

[91] Kinoshita Y, Kalir T, Dottino P, Kohtz DS (2012). Nuclear distributions of NUP62 and NUP214 suggest architectural diversity and spatial patterning among nuclear pore complexes. PLoS One.

[92] Guan T (1995). Structural analysis of the p62 complex, an assembly of O-linked glycoproteins that localizes near the central gated channel of the nuclear pore complex. Mol. Biol. Cell.

[93] Chou C-C (2018). TDP-43 pathology disrupts nuclear pore complexes and nucleocytoplasmic transport in ALS/FTD. Nat. Neurosci..

[94] Saberi S (2018). Sense-encoded poly-GR dipeptide repeat proteins correlate to neurodegeneration and uniquely co-localize with TDP-43 in dendrites of repeat-expanded C9orf72 amyotrophic lateral sclerosis. Acta Neuropathol..

[95] Cook, C. N. et al. C9orf72 poly(GR) aggregation induces TDP-43 proteinopathy. *Sci. Transl. Med*. **12**, eabb3774 (2020). 10.1126/scitranslmed.abb3774 PMC7989020 32878979

[96] McGurk L (2018). Poly(ADP-Ribose) Prevents Pathological Phase Separation of TDP-43 by Promoting Liquid Demixing and Stress Granule Localization. Mol. Cell.

[97] Gasset-Rosa F (2019). Cytoplasmic TDP-43 De-mixing Independent of Stress Granules Drives Inhibition of Nuclear Import, Loss of Nuclear TDP-43, and Cell Death. Neuron.

[98] Mann JR (2019). RNA Binding Antagonizes Neurotoxic Phase Transitions of TDP-43. Neuron.

[99] Chen Y, Cohen TJ (2019). Aggregation of the nucleic acid-binding protein TDP-43 occurs via distinct routes that are coordinated with stress granule formation. J. Biol. Chem..

[100] Buchan JR, Parker R (2009). Eukaryotic stress granules: the ins and outs of translation. Mol. Cell.

[101] Wu J (2013). Caspase-mediated cleavage of C53/LZAP protein causes abnormal microtubule bundling and rupture of the nuclear envelope. Cell Res.

[102] Yamakawa M (2015). Characterization of the dipeptide repeat protein in the molecular pathogenesis of c9FTD/ALS. Hum. Mol. Genet.

[103] Sakae N (2018). Poly-GR dipeptide repeat polymers correlate with neurodegeneration and Clinicopathological subtypes in C9ORF72-related brain disease. Acta Neuropathol. Commun..

[104] Kramer NJ (2018). CRISPR-Cas9 screens in human cells and primary neurons identify modifiers of C9ORF72 dipeptide-repeat-protein toxicity. Nat. Genet..

[105] Mizielinska S (2014). C9orf72 repeat expansions cause neurodegeneration in Drosophila through arginine-rich proteins. Science.

[106] Toyama BH (2013). Identification of long-lived proteins reveals exceptional stability of essential cellular structures. Cell.

[107] D’Angelo MA, Raices M, Panowski SH, Hetzer MW (2009). Age-dependent deterioration of nuclear pore complexes causes a loss of nuclear integrity in postmitotic cells. Cell.

[108] Verdone BM (2022). A mouse model with widespread expression of the C9orf72-linked glycine-arginine dipeptide displays non-lethal ALS/FTD-like phenotypes. Sci. Rep..

[109] Anderson, E. N. et al. Traumatic injury compromises nucleocytoplasmic transport and leads to TDP-43 pathology. *Elife* **10**, e67587 (2021). 10.7554/eLife.67587 PMC8169113 34060470

[110] Pandey UB (2007). HDAC6 rescues neurodegeneration and provides an essential link between autophagy and the UPS. Nature.

[111] Al-Sarraj S (2011). p62 positive, TDP-43 negative, neuronal cytoplasmic and intranuclear inclusions in the cerebellum and hippocampus define the pathology of C9orf72-linked FTLD and MND/ALS. Acta Neuropathol..

[112] Mann DMA (2013). Dipeptide repeat proteins are present in the p62 positive inclusions in patients with frontotemporal lobar degeneration and motor neurone disease associated with expansions in C9ORF72. Acta Neuropathol. Commun..

[113] Otte CG (2020). Optogenetic TDP-43 nucleation induces persistent insoluble species and progressive motor dysfunction in vivo. Neurobiol. Dis..

[114] Lin Y-C (2021). Interactions between ALS-linked FUS and nucleoporins are associated with defects in the nucleocytoplasmic transport pathway. Nat. Neurosci..

[115] Kim HJ, Taylor JP (2017). Lost in transportation: nucleocytoplasmic transport defects in ALS and other neurodegenerative diseases. Neuron.

[116] Kinoshita Y (2009). Nuclear contour irregularity and abnormal transporter protein distribution in anterior horn cells in amyotrophic lateral sclerosis. J. Neuropathol. Exp. Neurol..

[117] Aizawa H, Yamashita T, Kato H, Kimura T, Kwak S (2019). Impaired Nucleoporins Are Present in Sporadic Amyotrophic Lateral Sclerosis Motor Neurons that Exhibit Mislocalization of the 43-kDa TAR DNA-Binding Protein. J. Clin. Neurol..

[118] Yamashita T, Aizawa H, Teramoto S, Akamatsu M, Kwak S (2017). Calpain-dependent disruption of nucleo-cytoplasmic transport in ALS motor neurons. Sci. Rep..

[119] Yang P (2020). G3BP1 Is a Tunable Switch that Triggers Phase Separation to Assemble Stress Granules. Cell.

[120] Kedersha N (2016). G3BP-Caprin1-USP10 complexes mediate stress granule condensation and associate with 40S subunits. J. Cell Biol..

[121] Bracha D (2018). Mapping local and global liquid phase behavior in living cells using photo-oligomerizable seeds. Cell.

[122] Van Treeck B (2018). RNA self-assembly contributes to stress granule formation and defining the stress granule transcriptome. Proc. Natl Acad. Sci. USA.

[123] Verdone BM (2021). A Mouse Model with Widespread Expression of the C9orf72-Linked Glycine-Arginine Dipeptide Displays Non-Lethal ALS/FTD-Like Phenotypes. Sci. Rep..

[124] Zhang K (2018). Stress granule assembly disrupts nucleocytoplasmic transport. Cell.

[125] Ziller MJ (2018). Dissecting the functional consequences of de novo DNA methylation dynamics in human motor neuron differentiation and physiology. Cell Stem Cell.

[126] Tanaka KF (2010). Flexible Accelerated STOP Tetracycline Operator-knockin (FAST): a versatile and efficient new gene modulating system. Biol. Psychiatry.

[127] Soriano P (1999). Generalized lacZ expression with the ROSA26 Cre reporter strain. Nat. Genet..

[128] Kisseberth WC, Brettingen NT, Lohse JK, Sandgren EP (1999). Ubiquitous expression of marker transgenes in mice and rats. Dev. Biol..

[129] Zambrowicz BP (1997). Disruption of overlapping transcripts in the ROSA beta geo 26 gene trap strain leads to widespread expression of beta-galactosidase in mouse embryos and hematopoietic cells. Proc. Natl Acad. Sci. USA.

[130] Kinoshita Y (2014). Role for NUP62 depletion and PYK2 redistribution in dendritic retraction resulting from chronic stress. Proc. Natl Acad. Sci. USA.

[131] Ni J-Q (2011). A genome-scale shRNA resource for transgenic RNAi in Drosophila. Nat. Methods.

[132] Casci I (2019). Muscleblind acts as a modifier of FUS toxicity by modulating stress granule dynamics and SMN localization. Nat. Commun..

[133] Ramesh N, Kour S, Anderson EN, Rajasundaram D, Pandey UB (2020). RNA-recognition motif in Matrin-3 mediates neurodegeneration through interaction with hnRNPM. Acta Neuropathol. Commun..

[134] Anderson EN (2018). Traumatic injury induces stress granule formation and enhances motor dysfunctions in ALS/FTD models. Hum. Mol. Genet.

[135] Schmittgen TD, Livak KJ (2008). Analyzing real-time PCR data by the comparative C(T) method. Nat. Protoc..

