# Supplementary material for: NUP62 localizes to ALS/FTLD pathological assemblies and contributes to TDP-43 insolubility
Source: Nat Commun. 2022 Jun 13;13:3380. doi: 10.1038/s41467-022-31098-6 (PMC9192689; doi:10.1038/s41467-022-31098-6)
Supplement: Supplementary file 1 — Supplementary Information [file 41467_2022_31098_MOESM1_ESM.pdf]

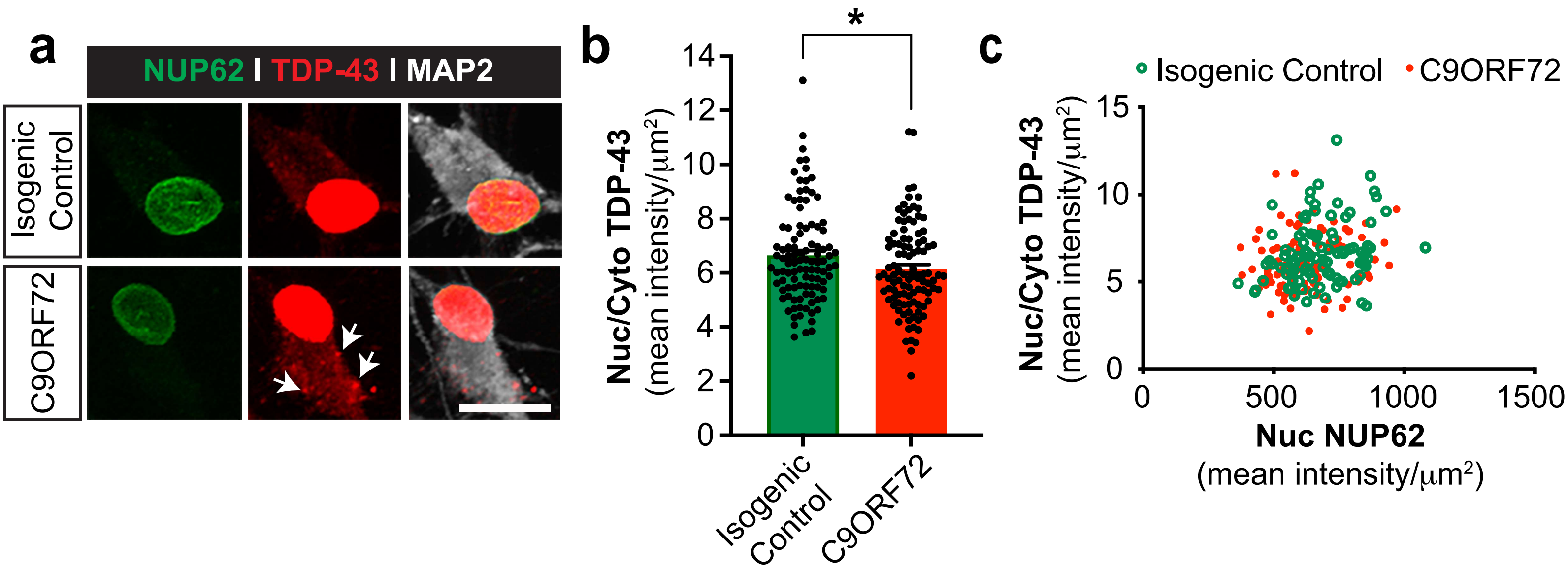
a
NUP62 I TDP-43 I MAP2
Isogenic Control
C9ORF72
b
*
Nuc/Cyto TDP-43
(mean intensity/μm²)
14
12
10
8
6
4
2
0
Isogenic Control
C9ORF72
c
Isogenic Control
C9ORF72
Nuc/Cyto TDP-43
(mean intensity/μm²)
15
10
5
0
0
500
1000
1500
Nuc NUP62
(mean intensity/μm²)

Supplementary Figure 1. TDP-43 mislocalization is not associated with abnormal nuclear NUP62 in C9-ALS iPSC neurons.

a) Isogenic control and C9-ALS iPSC neurons were immunostained for NUP62, TDP-43 and MAP2. Arrows show areas of increased cytoplasmic TDP-43. Scale bar: 10 μm **b)** Isogenic control and C9-ALS iPSC neurons detected by confocal microscopy following immunofluorescent staining. Nuclear-cytoplasmic (Nuc/Cyto) TDP43 levels were identified in maximum projection images. Analysis was conducted by region of interest (ROI) labeling of nuclear and cytoplasmic compartment. Quantification is presented as mean +/- SEM n=102 (C9-ALS) -103 (Isogenic Control) neurons per group. Statistical significance: unpaired two-tailed student's t-test * $p \leq 0.05$ **c)** Nuclear (Nuc) NUP62 levels and Nuclear-Cytoplasmic (Nuc/Cyto) TDP-43 were determined for isogenic control and C9-ALS iPSC neurons from maximum intensity projection confocal images. Values were then plotted for each individual neuron. Pearson's Correlation Analysis was conducted. There was a no correlation between nuclear NUP62 and nuc/cyto TDP-43 in isogenic control [r(101) = .24, p = .02] and C9-ALS [r(100) = .12, p = .22] neurons.

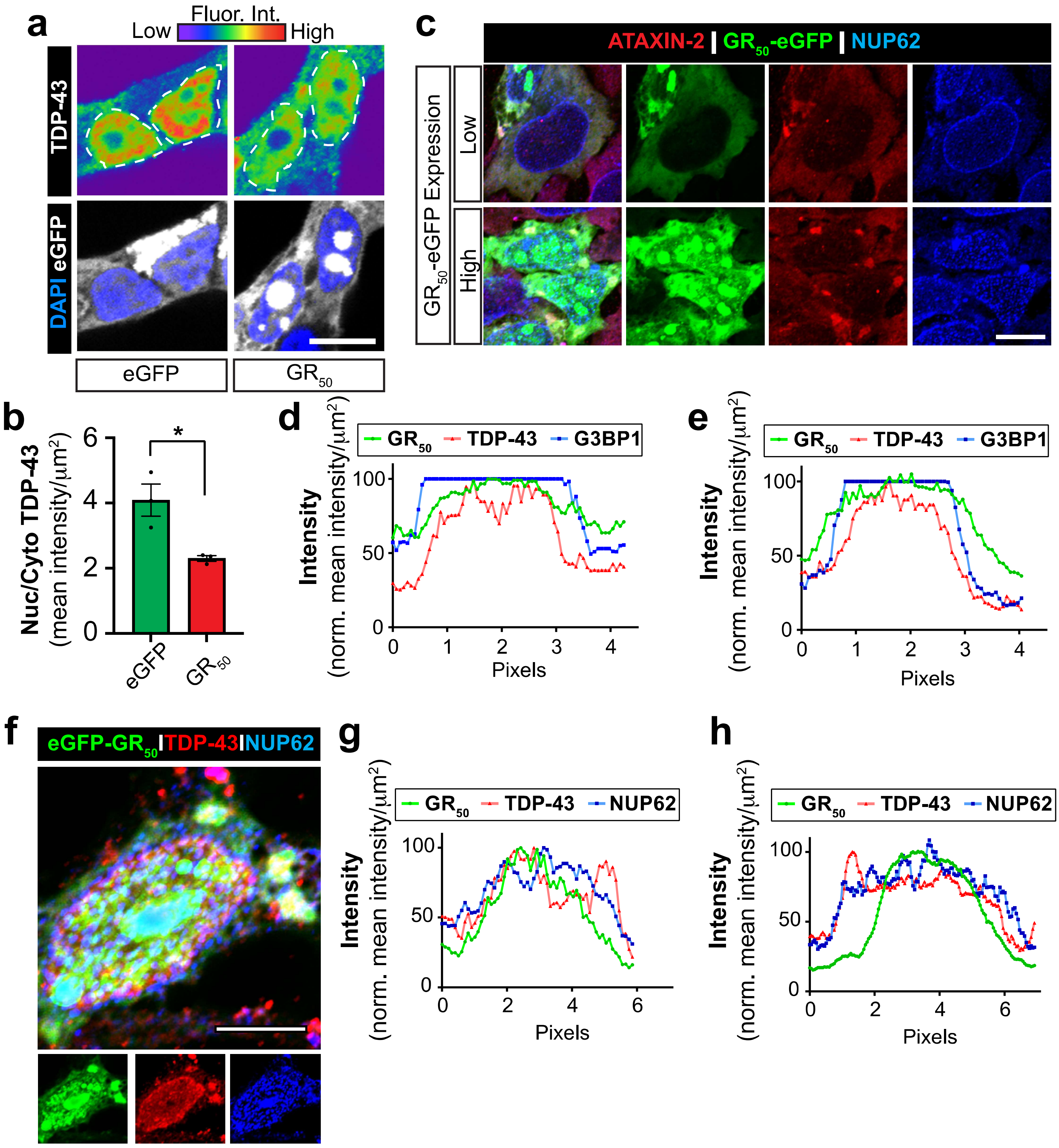
a
Fluor. Int.
Low
High
TDP-43
DAPI eGFP
eGFP
GR50
b
Nuc/Cyto TDP-43
(mean intensity/μm²)
*
eGFP
GR50
c
ATAXIN-2 | GR50-eGFP | NUP62
GR50-eGFP Expression
Low
High
d
GR50
TDP-43
G3BP1
Intensity
(norm. mean intensity/μm²)
Pixels
e
GR50
TDP-43
G3BP1
Intensity
(norm. mean intensity/μm²)
Pixels
f
eGFP-GR50|TDP-43|NUP62
g
GR50
TDP-43
NUP62
Intensity
(norm. mean intensity/μm²)
Pixels
h
GR50
TDP-43
NUP62
Intensity
(norm. mean intensity/μm²)
Pixels

Supplementary Figure 2. Poly-GR induces TDP-43 mislocalization to NUP62 cytoplasmic assemblies.

a) Image depicting TDP-43 distribution in HEK293 cells expressing GR_{50}-eGFP or eGFP control plasmid DNA. TDP-43 immunostaining is presented in spectral colors. Warmer colors represent higher TDP-43 levels whiles cooler colors show lower TDP-43 levels. DAPI is presented in blue and eGFP or GR_{50}-eGFP constructs are shown in white. **b)** Quantification of nuclear and cytoplasmic distribution of TDP-43 signal from cell in confocal images represented in Supplementary Figure 2A. Regions of interest (ROIs) were drawn around DAPI or cytoplasmic signal to determine TDP-43 in their respective regions. Nuclear area was determined according to $DAPI^+$ staining and cytoplasmic region was determined by non-nuclear eGFP signal. Each symbol represents biologically independent sample with a total of 60 (eGFP) or 105 (GR_{50}) cells analyzed. Data are shown as mean +/- SEM**. c)** HEK293 cells were transfected with 200 ng GR_{50}-eGFP plasmid DNA for 24h and immunostained for SG marker ATAXIN-2 and NUP62. Cells expressing low (top row) and high (bottom row) levels of GR_{50}-eGFP are represented. We observe the formation of Ataxin-2^+ accumulations with high GR_{50}-eGFP expression levels. NUP62 is detected in cytoplasmic Ataxin-2^+ condensates. **d-e)** Intensity profile plots of additional cytoplasmic GR_{50}-eGFP condensates. An intensity profile plot line was drawn through the condensate and signal intensity is plotted across the length of line. This data further supports the hypothesis that GR_{50}-eGFP, G3BP1, and TDP-43 exist within the same space. **f)** GR_{50}-eGFP transfected HEK293 cells were also immunostained for TDP-43 and NUP62. Maximum intensity projection confocal image reveals colocalization of TDP-43 and NUP62 in cytoplasmic GR_{50}-eGFP condensates. Top image is a merge of all channels and individual signals are presented below. **g-h)** Intensity profile plots of additional cytoplasmic GR_{50}-eGFP condensates with TDP-43 and NUP62. An intensity profile plot line was drawn through the condensate and signal intensity is plotted across the length of line. This data further supports the hypothesis that GR_{50}-eGFP,

TDP-43 and NUP62 exist within the same space. Statistically significant difference between control and GR_{50} expression groups was determined by two-tailed, unpaired t-test. * $p \leq 0.05$ vs control. Scale bar: 10 μm

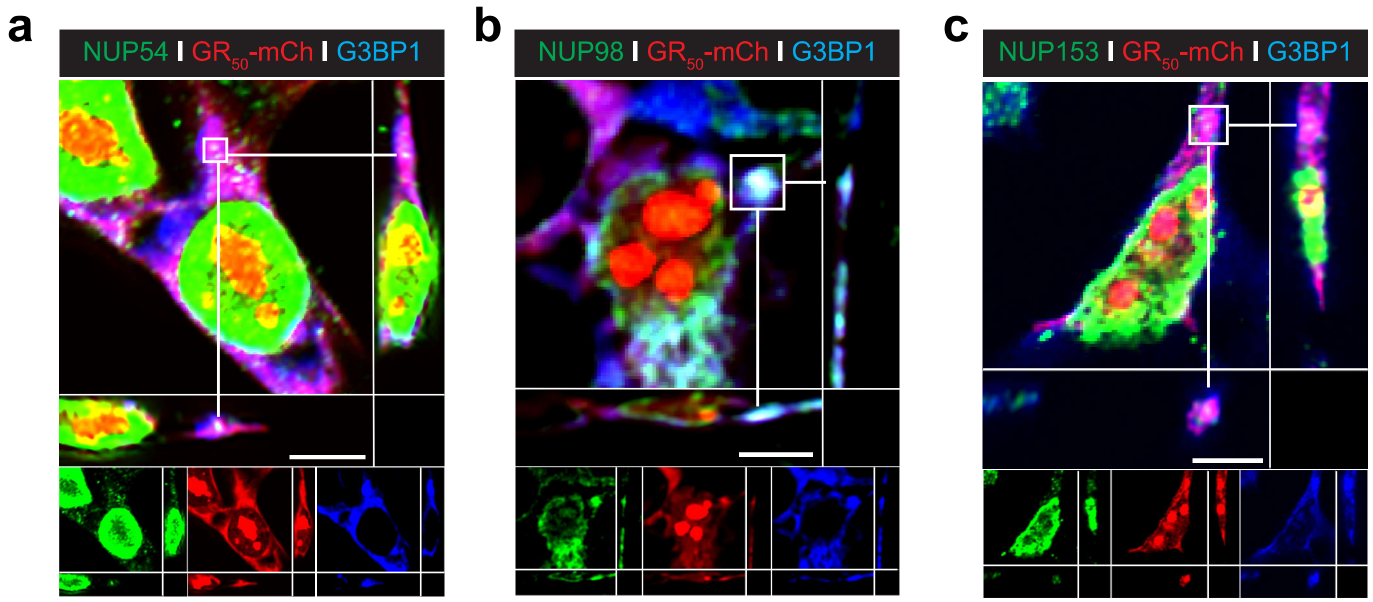

a
NUP54 | GR50-mCh | G3BP1
b
NUP98 | GR50-mCh | G3BP1
c
NUP153 | GR50-mCh | G3BP1

Supplementary Figure 3. Poly-GR condensates sequester FG nucleoporins

HEK293 cells were transfected with GR_{50}-mCh. FG nucleoporins NUP54, NUP98 and NUP153 were then detected by immunofluorescent staining. Orthogonal view images were processed by automatic 3D deconvolution and show the presence of FG nucleoporins in GR_{50}-mCh: G3BP1 structures. **a)** NUP54 **b)** NUP98 **c)** NUP153. Scale bar: 10 μm

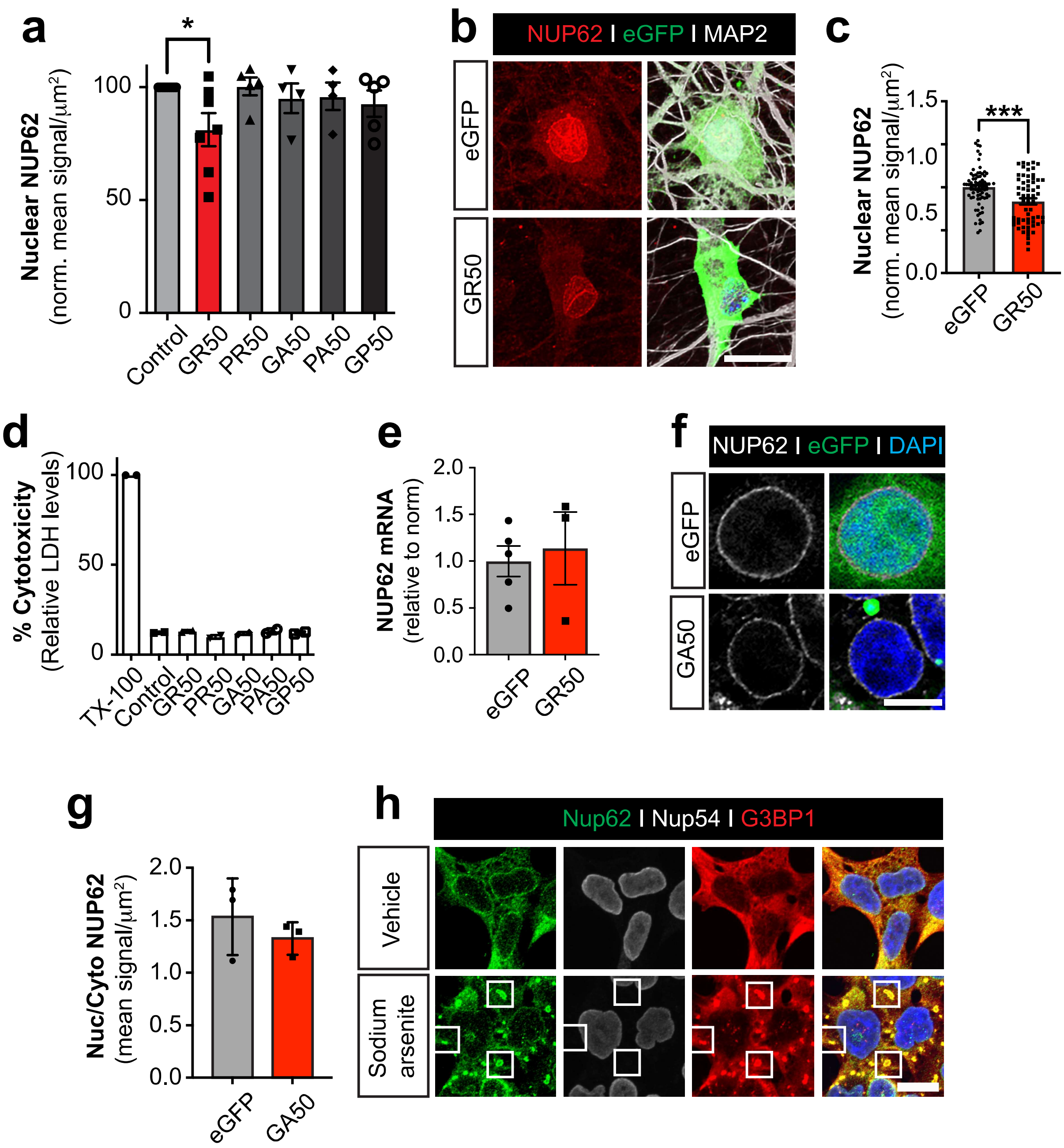

a
*
Nuclear NUP62
(norm. mean signal/µm²)
100
50
0
Control
GR50
PR50
GA50
PA50
GP50
b
NUP62 I eGFP I MAP2
eGFP
GR50
c
Nuclear NUP62
(norm. mean signal/µm²)
1.5
1.0
0.5
0.0
***
eGFP
GR50
d
% Cytotoxicity
(Relative LDH levels)
100
50
0
TX-100
Control
GR50
PR50
GA50
PA50
GP50
e
NUP62 mRNA
(relative to norm)
2.0
1.5
1.0
0.5
0.0
eGFP
GR50
f
NUP62 I eGFP I DAPI
eGFP
GA50
g
Nuc/Cyto NUP62
(mean signal/µm²)
2.0
1.5
1.0
0.5
0.0
eGFP
GA50
h
Nup62 I Nup54 I G3BP1
Vehicle
Sodium arsenite

Supplementary Figure 4. Poly-GR causes nuclear NUP62 loss at subtoxic concentrations.

a) Quantification of nuclear NUP62 levels by immunofluorescence staining in HEK293 cells expressing Control (eGFP or mCh), GR_{50}, PR_{50}, GA_{50}, PA_{50}, or GP_{50}. . Symbols represent biologically independent experiments. Sample size: Control (n=9), GR_{50} (n=7), PR_{50} (n=5), GA_{50} (n=4), PA_{50} (n=4), or GP_{50} (n=5). Representative images of Supplementary Figure 2a data is shown in Figure 3. Statistical significance was determined by one-way ANOVA with Dunnett's multiple comparison test. * $p \leq 0.05$ vs control. Data are shown as mean +/- SEM. **b)** Control iPSC neurons were infected with lentiviral GR_{50}-eGFP. Immunofluorescent staining for NUP62 and MAP2 was then conducted prior to confocal microscopy. Images shows are maximum intensity projections. Scale bar: 20 μm **c)** Nuclear NUP62 levels were measured by region of interest (ROI) selection of $DAPI^+$ signal from maximum intensity projection images that were obtained by confocal microscopy. Quantification reveals mean signal +/- SEM. Statistically significant difference between control and GR_{50}-eGFP expression groups was determined by two-tailed, unpaired t-test. *** $p \leq 0.001$ vs control. n= 75 (eGFP) and 61 (GR_{50}) $MAP2^+$ neurons collected from two separate experiments. **d)** Cell death was measured by LDH cytotoxicity assay in HEK293 cells expressing the DPR construct for 24 h. TX-100-treated HEK293 cells were included during assay as a positive measure of cell death. No significant increase in cell death was observed in cells expressing dipeptide repeat proteins relative to Control group. Symbols indicate biologically independent experiments. n=2 biologically independent experiments. **e)** NUP62 transcript levels were measured in HEK293 cells after 24h of transfection with control (eGFP) or GR_{50}-eGFP plasmid DNA and showed no significant difference between groups. Normalization of gene levels were expressed relative to GAPDH transcript levels. Data are shown as mean +/- SD. n=3 biologically independent experiments. **f)** Representative images of HEK293 cells transfected with control (eGFP) or GA_{50}-eGFP. Cells were then immunostained for NUP62 and images captured by confocal microscopy. Scale bar: 20 μm **g)** Quantification of NUP62 distribution

following control or GA_{50} plasmid expression as presented in subpanel F. Regions of interest (ROI) were drawn around nucleus and cytoplasm to determine NUP62 intensity in each compartment. Nuc/Cyto NUP62 distribution was then calculated for each cell and is plotted as the average +/- SD. No statistically significant difference was determined by two-tailed, unpaired t-test. Three biologically independent experiments. **h)** HEK293 cells were treated with vehicle (top row) or sodium arsenite (bottom row). Immunofluorescent staining for NUP62, NUP54 and G3BP1 show NUP62 colocalization with $G3BP1^+$ stress granules but an absence of NUP54:G3BP1 colocalization. n=75 (vehicle) or 100 (sodium arsenite) cells. Scale bar: 20 μm

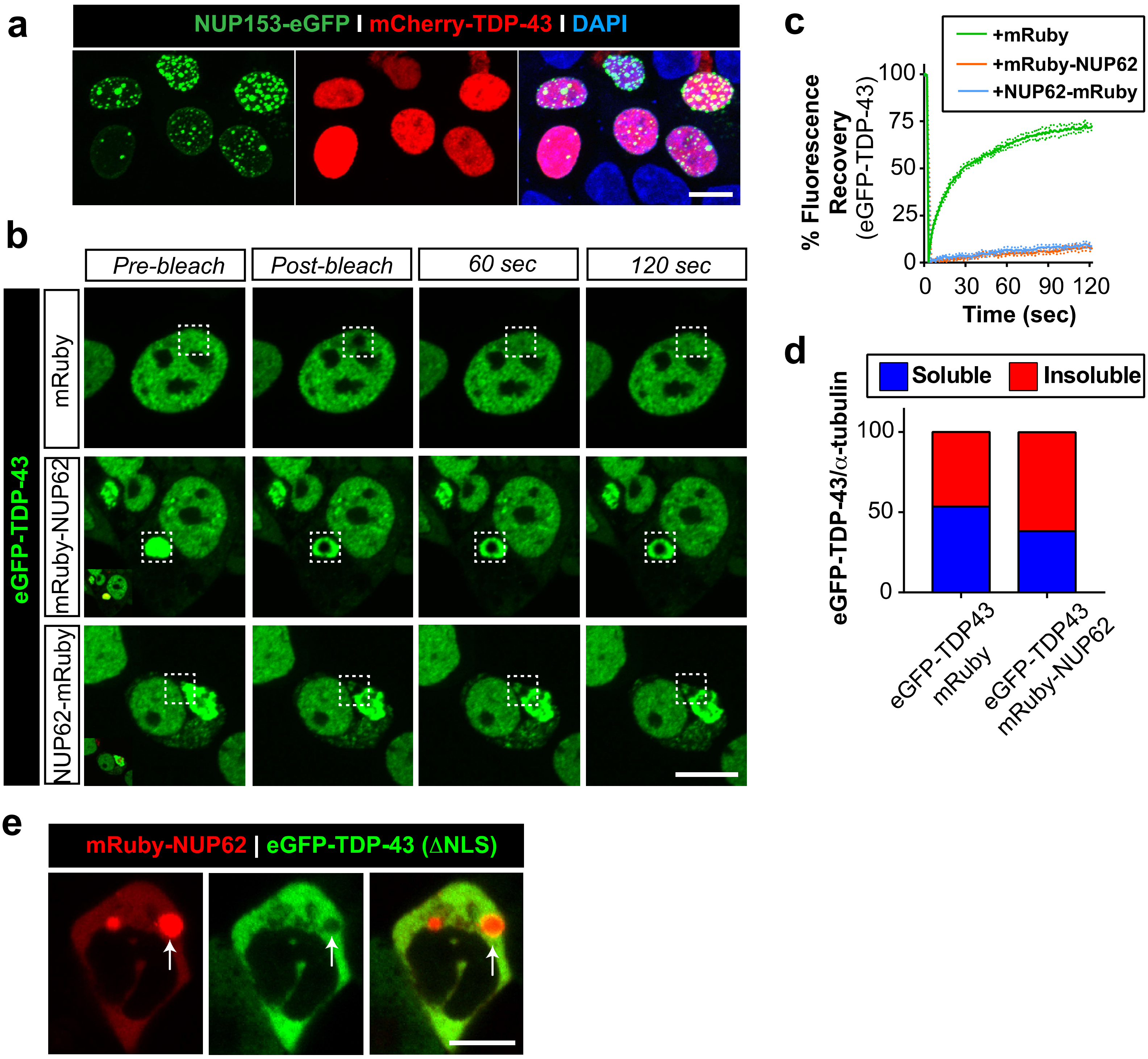
a
NUP153-eGFP | mCherry-TDP-43 | DAPI
b
Pre-bleach
Post-bleach
60 sec
120 sec
eGFP-TDP-43
mRuby
mRuby-NUP62
NUP62-mRuby
c
+mRuby
+mRuby-NUP62
+NUP62-mRuby
% Fluorescence Recovery (eGFP-TDP-43)
100
75
50
25
0
0
30
60
90
120
Time (sec)
d
Soluble
Insoluble
eGFP-TDP-43/α-tubulin
100
50
0
eGFP-TDP43 mRuby
eGFP-TDP43 mRuby-NUP62
e
mRuby-NUP62 | eGFP-TDP-43 (ΔNLS)

Supplementary Figure 5. mRuby-NUP62 cytoplasmic droplets promote eGFP-TDP-43 insolubility

a) Representative image of HEK293 cells co-transfected with NUP153-eGFP and mCherry-TDP-43. Scale bar: 10 µm**.** Similar results were observed in two independent experiments. **b)** HEK293 cells were co-transfected with eGFP-TDP-43 and mRuby, mRuby-NUP62, or NUP62-mRuby to determine whether both orientations of fluorescently labelled NUP62 protein form insoluble, cytoplasmic TDP-43 structures. Representative FRAP analysis images of eGFP-TDP43 are shown. White box indicates region of FRAP. Scale bar: 10 µm **c)** Quantification of FRAP analysis shows both mRuby-NUP62 and NUP62-mRuby reduce eGFP-TDP43 fluorescent signal recovery. Data are shown as mean +/- SD. **d)** Quantification of soluble and insoluble eGFP-TDP43 by western blot analysis. Similar results were observed across three independent experiments. Representative blot is shown in Figure 5. **e)** HEK293 cells were co-transfected with mRuby-NUP62 and eGFP-TDP43 with NLS mutation point mutations (ΔNLS)[137]. Cytoplasmic mRuby-NUP62 condensates do not colocalize with eGFP-TDP43 (ΔNLS). These observations were detected in two independent experiments**.** Scale bar: 10 µm.

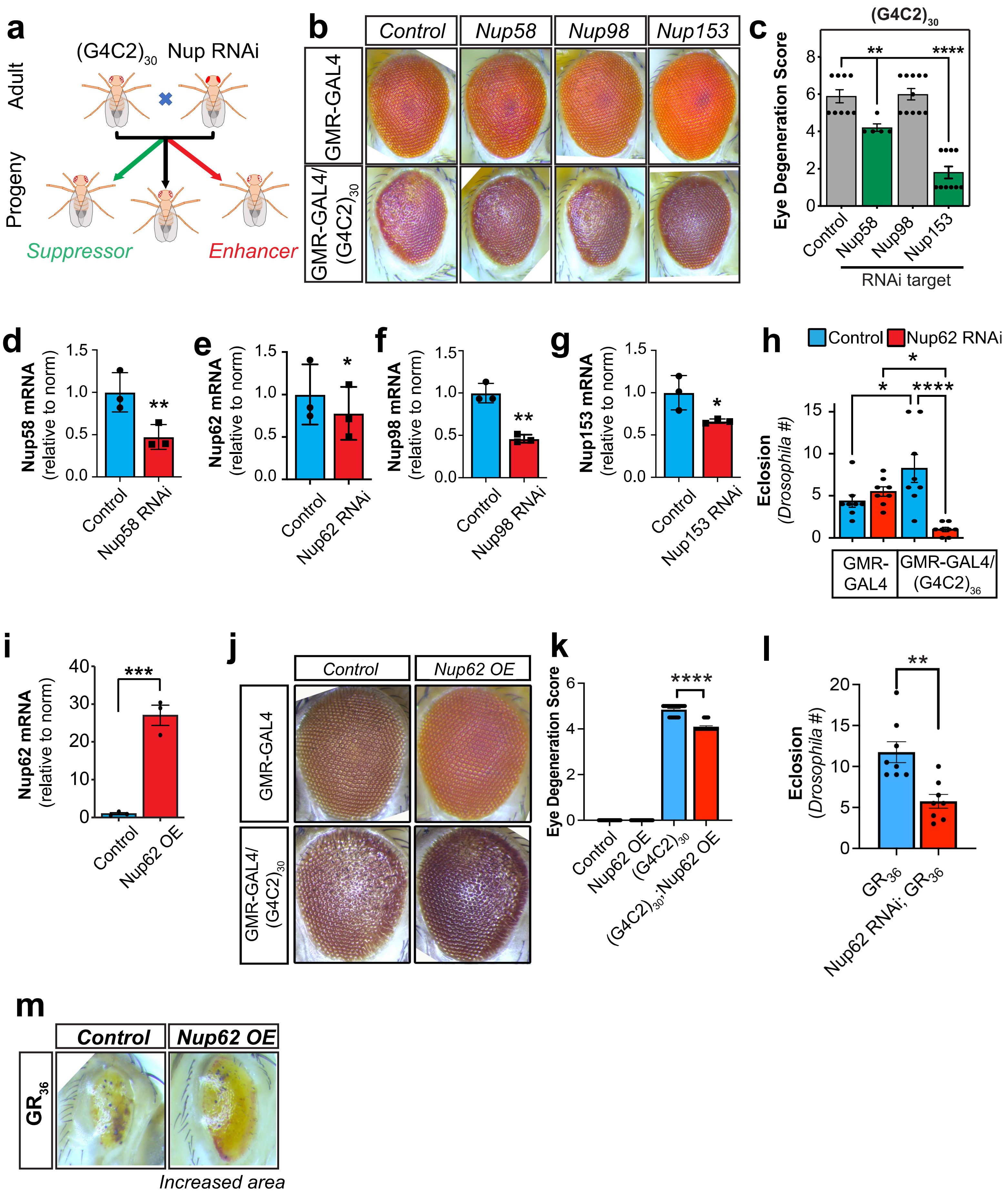
a
(G4C2)30 Nup RNAi
Adult
Progeny
Suppressor
Enhancer
b
Control
Nup58
Nup98
Nup153
GMR-GAL4
GMR-GAL4/ (G4C2)30
c
(G4C2)30
Eye Degeneration Score
**
****
Control
Nup58
Nup98
Nup153
RNAi target
d
Nup58 mRNA (relative to norm)
**
Control
Nup58 RNAi
e
Nup62 mRNA (relative to norm)
*
Control
Nup62 RNAi
f
Nup98 mRNA (relative to norm)
**
Control
Nup98 RNAi
g
Nup153 mRNA (relative to norm)
*
Control
Nup153 RNAi
h
Control
Nup62 RNAi
Eclosion (Drosophila #)
*
*
****
GMR-GAL4
GMR-GAL4/ (G4C2)36
i
Nup62 mRNA (relative to norm)
***
Control
Nup62 OE
j
Control
Nup62 OE
GMR-GAL4
GMR-GAL4/ (G4C2)30
k
Eye Degeneration Score
****
Control
Nup62 OE
(G4C2)30
(G4C2)30;Nup62 OE
l
Eclosion (Drosophila #)
**
GR36
Nup62 RNAi; GR36
m
Control
Nup62 OE
GR36
Increased area

Supplementary Figure 6. Validation of studies examining Nup62 as a genetic modifier of C9-ALS/FTD *Drosophila* models.

a) This schematic depicts Drosophila genetic modifier studies. A reduction in progeny eye degeneration by Nup RNAi is classified as a genetic suppressor while enhanced progeny eye degeneration is identified as genetic enhancers. **b)** Representative images of fly eyes from GMR-GAL4 wild-type (top row) or $(G4C2)_{30}$ repeat expansion (bottom row) flies combined with control (eGFP) or UAS-FG nup (Nup58, Nup98, Nup153) RNAi flies. **c)** Quantification of scored fly eye degeneration. Nup58 and Nup153 RNAi significantly reduces retinal degeneration of $(G4C2)_{30}$ repeat expansion. However, Nup98 RNAi does not alter $(G4C2)_{30}$ repeat expansion-mediated retinal degeneration. Symbols are indicative of eye degeneration score for individual flies evaluated. Statistically significant differences in eclosion frequency were determined by one-way ANOVA with Dunnett's multiple comparison's test: ** $p \leq 0.01$, **** $p \leq 0.0001$. Data are shown as mean +/- SEM. **d-g)** mRNA was extracted from Drosophila fly heads and qPCR analysis confirmed downregulation of FG Nup mRNA levels. A minimum of 9 fly heads were pulled together for each group and run-in triplicate technical replicates. Normalization of gene levels were expressed relative to alpha-tubulin. Statistically significant differences between control and RNAi fly lines were determined by one-tailed, paired student's t-test * $p \leq 0.05$; ** $p \leq 0.01$ vs control. W1118 was the control group for Nup58, Nup98, and Nup153 RNAi validation experiments and GMR-GAL4 x UAS-eGFP was the control group for Nup62 RNAi validation experiments. **h)** Bar graph shows the raw quantification for $(G4C2)_{36}$ repeat expansion fly eclosion in the presence and absence of Nup62 RNAi. The bars show average eclosion per day over the course of eight days while individual dots are representative of fly counts for one 24 h eclosion period. $(G4C2)_{36}$;GMR-GAL4 line crossed with UAS-eGFP fly line was used as the control group. Samples sizes n=8 flies per group. Statistically significant differences in eclosion frequency were determined by one-way ANOVA with Tukey's multiple comparison's test: * $p \leq 0.05$, **** $p \leq$

0.0001. Data are shown as mean +/- SEM. **i)** mRNA was extracted from Drosophila fly heads and qPCR analysis confirmed increased Nup62 transcript levels in the Nup62 overexpression fly (Nup62 OE). A minimum of 9 fly heads were pulled together for each group and run-in triplicate technical replicates. Normalization of gene levels were expressed relative to alpha-tubulin. Statistically significant differences between control and Nup62 overexpression fly lines were determined by unpaired two-tailed student's t-test *** $p \leq 0.001$ vs W1118 control. Data are shown as mean +/- SEM. **j)** Representative images of fly eyes from GMR-GAL4 wild-type (top row) or GMR-GAL4/$(G4C2)_{30}$ repeat (bottom row) flies combined with control (W1118) or UAS-Nup62 OE. Images were taken within 24h of eclosion. **k)** Quantification of scored fly eye degeneration that was assessed within 24h of eclosion. Statistically significant differences were determined by one-way ANOVA with Tukey's multiple comparison's test: **** $p \leq 0.0001$. Samples size n=22 (for each Control, Nup62 OE, $(G4C2)_{30}$) or n=21 ($(G4C2)_{30}$;Nup62 OE flies per group). **l)** Quantification of fly eclosion for GR_{36} and Nup62 RNAi;GR_{36}. The bars show average eclosion per day over the course of eight days while individual dots are representative of fly counts for one 24 h eclosion period. Statistically significant differences in eclosion frequency were determined by unpaired two-tailed student's t-test: ** $p \leq 0.01$ n=8 flies per group. **m)** Combination GMR-GAL4/TM3; Nup62 OE/Sb were crossed with UAS-$(GR)_{36}$ flies. Progeny were collected within 24h of eclosion and evaluated for fly eye degeneration. In the absence of Nup62 overexpression, the $(GR)_{36}$ progeny eyes show largely reduced eye size and an absence of ommatidial organization. However, Nup62 overexpression attenuates this effect. W1118 phenotype *Drosophila* were used as control. Data are shown as mean +/- SEM.

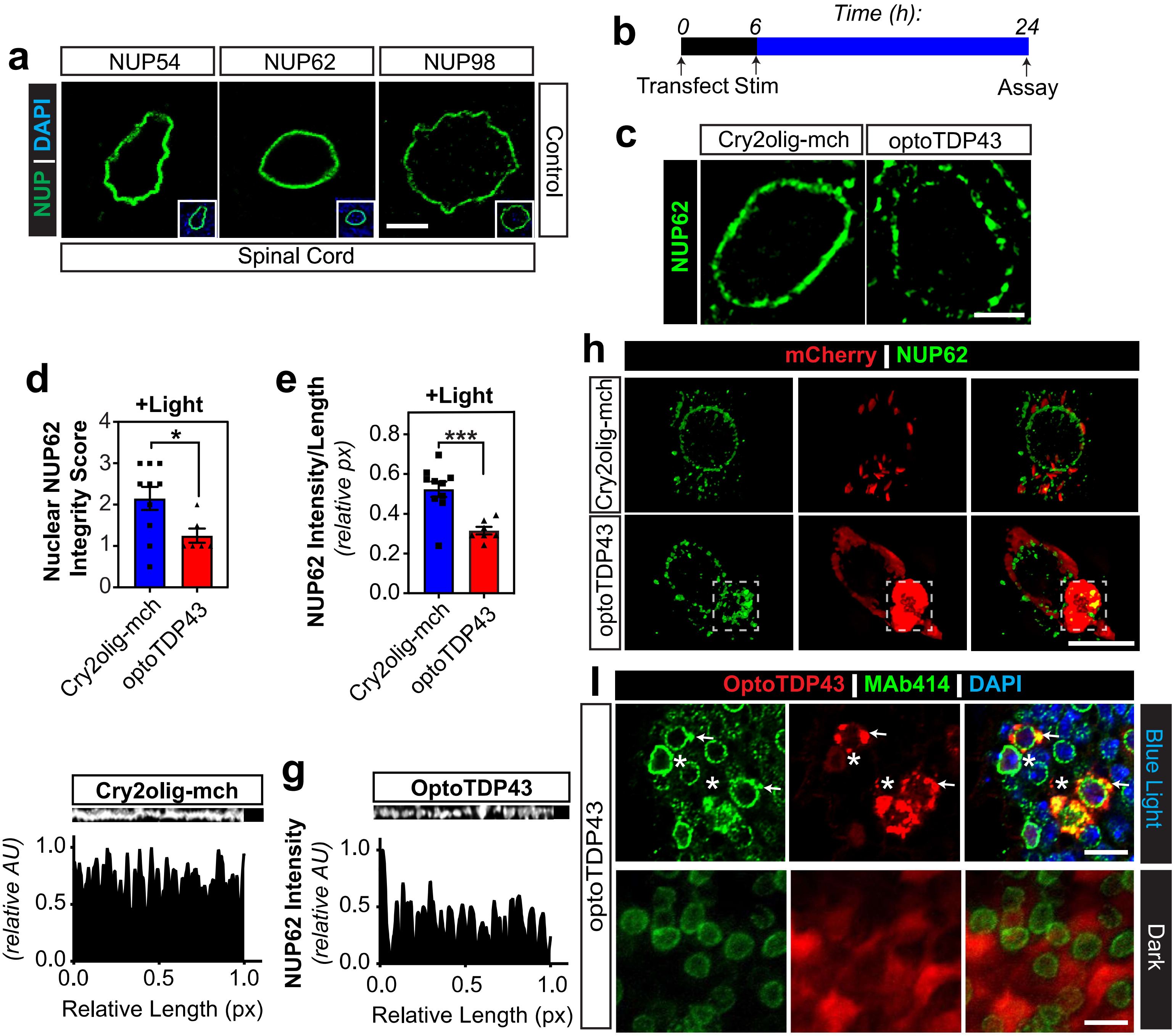
a
NUP54
NUP62
NUP98
NUP | DAPI
Control
Spinal Cord
b
Time (h):
0
6
24
Transfect Stim
Assay
c
Cry2olig-mch
optoTDP43
NUP62
d
+Light
Nuclear NUP62 Integrity Score
0
1
2
3
4
*
Cry2olig-mch
optoTDP43
e
+Light
NUP62 Intensity/Length
(relative px)
0.0
0.2
0.4
0.6
0.8
***
Cry2olig-mch
optoTDP43
f
Cry2olig-mch
NUP62 Intensity
(relative AU)
0.0
0.5
1.0
Relative Length (px)
g
OptoTDP43
NUP62 Intensity
(relative AU)
0.0
0.5
1.0
Relative Length (px)
h
mCherry | NUP62
Cry2olig-mch
optoTDP43
i
OptoTDP43 | MAb414 | DAPI
optoTDP43
Blue Light
Dark

Supplementary Figure 7. FG nups localize to OptoTDP43 inclusions

a) NUP54, NUP62, and NUP98 staining in the spinal cord of control post-mortem tissue. **b)** Schematic describing *in vitro* blue-light stimulation paradigm. HEK293 cells were transfected with OptoTDP43 or Cry2-Olig control constructs prior to 18 h of blue light exposure. **c)** Representative single slice (0.125 μm) N-SIM Super-resolution microscopy images NUP62 immunofluorescent staining (green) in HEK293 cells. OptoTDP43$^+$ cells exhibited the appearance of more fragmented NUP62 staining around nucleus. **d)** Nuclear NUP62 integrity in N-SIM Super-resolution images (n=10 (Cry2olig-mch)- 6 (optoTDP43) cells/group) was scored by blinded, unbiased observer. Scores are described in Supplementary Table 4. OptoTDP43$^+$ cells received lower nuclear NUP62 integrity scores relative to control. **e)** Nuclear NUP62 fragmentation quantified according to intensity profile plots. Area under the curve (AUC) for NUP62 signal was averaged and expressed as a function of nuclear NUP62 circumference to account for variability in nuclear size (n=10 (Cry2olig-mch)- 7 (optoTDP43) cells/group). **f-g)** Representative raw nuclear fragmentation data from control (Cry2-olig) and OptoTDP43 expressing cells shown in Supplementary Figure 7C and average data is graphed in Supplementary Figure 7E. **h)** N-SIM Super-resolution images of Control (Cry2-olig; top row) and OptoTDP43$^+$ cells immunostained for NUP62 (green). Images revealed NUP62 sequestration into OptoTDP43 aggregates and this is highlighted by white box. Red signal depicts mCherry tag that is present on Cry2-Oligo and OptoTDP43 constructs. **i)** Representative image of FG NUPs being sequestered into optoTDP43 aggregates in Optofly model. FG NUPs, such as NUP62, were detected by MAb414 antibody in the ventral nerve cord of third instar larvae after 24h of blue-light stimulation (top row) or darkness (bottom row). Arrows highlight optoTDP43 aggregates that co-stain for MAb414 while asterisks indicated optoTDP43 aggregates that are FG nup negative. Similar results were observed in two biologically independent experiments. Statistical significance was determined by unpaired, two-tailed t-test. *

$p \leq 0.05$, *** $p \leq 0.001$ vs control. Scale bar: 2.5 µm (C), 10 µm (A, H & I) Data are shown as mean +/- SEM.

Supplementary Table 1. Postmortem Human Tissue Cases

Case ID	Figure ID	Figure location	Diagnostic Classification	Sex	Age
CW98-095	C9-ALS + FTD Patient 1	Figure 1A-B, 7A-C	fALS (C9ORF72) with FTLD-TDP43	M	75
CW13-097	C9-ALS + FTD Patient 2	Figure 1A-B, 7B-C	fALS (C9ORF72) with FTLD-TDP43	F	62
CW16-098	sALS 1	Figure 7D-E	sALS	M	60
CW05-096	sALS + FTLD	Figure 7F	sALS with FTLD-TDP43	M	57
CW13-099	sALS 2	Figure 7F	sALS with mild nonmotor TDP43 pathology	F	74
CW00-29	FTLD-FUS 1	Figure 7G	FTLD-FUS	M	37
CW05-004	FTLD-FUS 2	Figure 7G	FTLD-FUS	M	48
CW99-100	Control	Sup Figure 7A	Control	M	54

Supplementary Table 2. Patient iPSC lines

iPSC Line ID	Figure ID	Genotype	Sex	Source
CS52iALS-C9n6 ISO	Isogenic Control (Figure 1D)	Isogenic corrected CS52	Male	Cedars Sinai iPSC Core
CS52iALS-n6A	C9ORF72 (Figure 1D)	C9ORF72	Male	Cedars Sinai iPSC Core
Con 18a	Control (Figure 1E-G)	Healthy control	Female	Boulting et al., 2011; Nat Biotech
Con 15_12	Control (Figure 1E-G)	Healthy control	Female	RUCDR Infinite Biologics
C9#2	C9ORF72 (Figure 1E-G)	C9ORF72	Female	RUCDR Infinite Biologics
C9 3.1	C9ORF72 (Figure 1E-G)	C9ORF72	Female	RUCDR Infinite Biologics
C9 10689	C9ORF72 (Figure 1E-G)	C9ORF72	Female	Coriell Institute for Medical Research

Supplementary Table 3. qPCR primers

Target	Species	Fwd Primer Sequence	Rev Primer Sequence
Nup58	Drosophila	CACTTTCTCCTTTGCCACCC	CCAGTTTCAGACCCGTGTTG
Nup62	Drosophila	TGCCGCCCAATCTTCAAACT	CGGTTGTTGGCAACTGG
Nup98	Drosophila	TTATGCGCCTGGCAGTTGTA	TGTCAAGTCAGCAGGAGTCG
Nup153	Drosophila	ATTGCGAAAGCAGTGTGTGC	TTGCTTTTGGCTTTGCCCTT
α-tubulin	Drosophila	CTTCCTCATCTTCCACTCGTTC	TGGTCTTGATGGTGGCAATAG
Nup62	Human	CTACAGGCGGGTTCACGTTT	CGGTGGAAGGGGTACTTGTG
GAPDH	Human	TCGGAGTCAACGGATTTG	GCCATGGGTGGAATCATATGG

Supplementary Table 4. Nuclear Integrity Scoring Criteria

Nuclear characteristics	Score
Good, clear obvious border	3
Minor disruptions, some same deformity	2
Large gaps, obvious shape irregularity	1
Absent nuclear border present	0

Supplementary Table 5. Cloning primers

Target	Base vector	Fwd Primer Sequence	Rev Primer Sequence
Nup62-mRuby	Nup62 (pcDNA3.1)	gcagtcgacggtaccgcgggcccggATGAGCGGGTTTAATTTTGGAGGCAC	ttagacaccatggtggcgaccggtgAGTCAAAGGTGATCCGGAAGCTGCG
mRuby-Nup62	Nup62-mRuby	gctgtacaagCGGGCCCGGATGAGCGGG	tgattatgatctagagtcgcTTAGTCAAAGGTGATCCGGAAGCTGCGC
Lenti-GR50	FLAG-GR50-eGFP	tttcttccatttcaggtgtcgtgacATGGATTACAAGGATGAC	acttcctctgccctctccactgcctTTACTTGTACAGCTCGTC

Supplementary Table 6

Case ID	Figure ID	Region	NUP62 + pTDP-43	NUP62 + FUS	NUP54 + pTDP43	NUP98 + p62
CW98-095	C9-ALS + FTD Patient 1	Hippocampus or mesial temporal cortex	+++++++<br>+	^	--	----++
CW98-095	C9-ALS + FTD Patient 1	Spinal cord	+++	^	^	^
CW13-097	C9-ALS + FTD Patient 2	Hippocampus or mesial temporal cortex	+++++++<br>+++++++<br>+++++++<br>+++	^	--	++++
CW13-097	C9-ALS + FTD Patient 2	Spinal cord	+++++	^	^	^
CW16-098	sALS 1	Spinal cord	++	^	---++	^
CW13-099	sALS 2	Hippocampus or mesial temporal cortex	++++++	^	--+	^
CW00-29	FTLD-FUS 1	Hippocampus	^	------------<br>-	^	^
CW05-004	FTLD-FUS 2	Hippocampus	^	------------<br>---------	^	^

Each symbol represents an inclusion found with the sample. (+: co-labeled for nucleoporin. -: absent of nucleoporin co-labelling.) Samples not characterized: ^
