# Supplementary material for: NUP62 localizes to ALS/FTLD pathological assemblies and contributes to TDP-43 insolubility
Source: Nat Commun. 2022 Jun 13;13:3380. doi: 10.1038/s41467-022-31098-6 (PMC9192689; doi:10.1038/s41467-022-31098-6)
Supplement: Supplementary file 2 — Description of Additional Supplementary Files [file 41467_2022_31098_MOESM2_ESM.pdf]

Description of Additional Supplementary Files

File Name: Supplementary Movie 1
Description: **Cytoplasmic mRuby-NUP62 condensates absent of eGFP-TDP-43 exhibit reversible condensate dynamics.** Timestamp shows imaging timeframe in hh:min:sec:msec and corresponds with Figure 5B schematic. mRuby-NUP62 is shown in red, and eGFP-TDP-43 is shown in green. Corresponding static images are shown in Figure 5A. Scale bar: 4 μm

File Name: Supplementary Movie 2
Description: **Cytoplasmic mRuby-NUP62:eGFP-TDP-43 condensates form irreversible structures.** Timestamp shows imaging timeframe in hh:min:sec:msec and corresponds with Figure 5B schematic. mRuby-NUP62 is shown in red, and eGFP-TDP-43 is shown in green. Corresponding static images are shown in Figure 5A. Scale bar: 4 μm

File Name: Supplementary Movie 3
Description: **mRuby-NUP62-eGFP-TDP-43 inclusion characterization by FRAP analysis.** Timestamp shows imaging timeframe in hh:min:sec:msec with photobleaching event at 2.83 sec. Corresponding static images are shown in Figure 5F (cytoplasmic condensate). Scale bar: 5 μm

File Name: Supplementary Movie 4
Description: **Nuclear eGFP-TDP-43 characterization by FRAP analysis to represent soluble control group.** Timestamp shows imaging timeframe in hh:min:sec:msec with photobleaching event at 2.83 sec. Corresponding static images are shown in Figure 5F (nuclear). Scale bar: 5 μm
