# Supplementary material for: NUP62 localizes to ALS/FTLD pathological assemblies and contributes to TDP-43 insolubility
Source: Nat Commun. 2022 Jun 13;13:3380. doi: 10.1038/s41467-022-31098-6 (PMC9192689; doi:10.1038/s41467-022-31098-6)
Supplement: Supplementary file 7 — Reporting Summary [file 41467_2022_31098_MOESM7_ESM.pdf]

nature research

Corresponding author(s): Christopher J. Donnelly
Last updated by author(s): Feb 4, 2022

Reporting Summary

Nature Research wishes to improve the reproducibility of the work that we publish. This form provides structure for consistency and transparency in reporting. For further information on Nature Research policies, see our Editorial Policies and the Editorial Policy Checklist.

Statistics

For all statistical analyses, confirm that the following items are present in the figure legend, table legend, main text, or Methods section.

n/a	Confirmed	
☐	☒	The exact sample size (*n*) for each experimental group/condition, given as a discrete number and unit of measurement
☐	☒	A statement on whether measurements were taken from distinct samples or whether the same sample was measured repeatedly
☐	☒	The statistical test(s) used AND whether they are one- or two-sided<br>*Only common tests should be described solely by name; describe more complex techniques in the Methods section.*
☒	☐	A description of all covariates tested
☐	☒	A description of any assumptions or corrections, such as tests of normality and adjustment for multiple comparisons
☐	☒	A full description of the statistical parameters including central tendency (e.g. means) or other basic estimates (e.g. regression coefficient) AND variation (e.g. standard deviation) or associated estimates of uncertainty (e.g. confidence intervals)
☐	☒	For null hypothesis testing, the test statistic (e.g. F, t, r) with confidence intervals, effect sizes, degrees of freedom and P value noted<br>*Give P values as exact values whenever suitable.*
☒	☐	For Bayesian analysis, information on the choice of priors and Markov chain Monte Carlo settings
☒	☐	For hierarchical and complex designs, identification of the appropriate level for tests and full reporting of outcomes
☐	☒	Estimates of effect sizes (e.g. Cohen's *d*, Pearson's *r*), indicating how they were calculated

Our web collection on statistics for biologists contains articles on many of the points above.

Software and code

Policy information about availability of computer code

Data collection	Nikon Elements AR Analysis 4.51 and ImageJ were used for image processing and analysis, GraphPad Prism Version 7 & 8 was used for data presentation and statistical analysis
Data analysis	Statistically significant differences between experimental groups were calculated by GraphPad Prism software and deemed significant when p ≤ 0.05. Statistically significant differences are described in the text or figure legends and were determined by unpaired Student's T-test when comparing two variables or one-way ANOVA with Dunnett or Tukey's multiple comparisons test when comparing multiple. Statistical analysis of nuclear Nup62 levels in control and C9orf72 ALS iPSC neurons was conducted by two-tailed Mann-Whitney test.

For manuscripts utilizing custom algorithms or software that are central to the research but not yet described in published literature, software must be made available to editors and reviewers. We strongly encourage code deposition in a community repository (e.g. GitHub). See the Nature Research guidelines for submitting code & software for further information.

Data

Policy information about availability of data

All manuscripts must include a data availability statement. This statement should provide the following information, where applicable:
- Accession codes, unique identifiers, or web links for publicly available datasets
- A list of figures that have associated raw data
- A description of any restrictions on data availability

All raw and processed data can be made available upon request.

Field-specific reporting

Please select the one below that is the best fit for your research. If you are not sure, read the appropriate sections before making your selection.

☒ Life sciences ☐ Behavioural & social sciences ☐ Ecological, evolutionary & environmental sciences

For a reference copy of the document with all sections, see nature.com/documents/nr-reporting-summary-flat.pdf

Life sciences study design

All studies must disclose on these points even when the disclosure is negative.

Sample size	Sample sizes are described in the methods, figure legends or main text.
Data exclusions	No data was excluded unless identified to be an outlier by ROUT method with Q equivalent to 1%.
Replication	Our studies employed several independent assays (Drosophila models, iPSC neurons, cellular expression systems, transgenic mouse model, post-mortem tissue) to replicate our findings. These studies were also a collaborative effort of five independent research groups.
Randomization	No randomization was used for any experiments in this manuscript
Blinding	Subjective assessment of nuclear Nup62 depletion by optoTDP43 was assess by an unbiased, blinded observer. In these studies, the images were de-identified to reduce subjectivity and bias. For experiments where experimental groups were apparent, image analysis software was used with identical configurations to eliminate any potential observer bias.

Reporting for specific materials, systems and methods

We require information from authors about some types of materials, experimental systems and methods used in many studies. Here, indicate whether each material, system or method listed is relevant to your study. If you are not sure if a list item applies to your research, read the appropriate section before selecting a response.

Materials & experimental systems

n/a	Involved in the study	
☐	☒	Antibodies
☐	☒	Eukaryotic cell lines
☒	☐	Palaeontology and archaeology
☐	☒	Animals and other organisms
☐	☒	Human research participants
☒	☐	Clinical data
☒	☐	Dual use research of concern

Methods

n/a	Involved in the study	
☒	☐	ChIP-seq
☒	☐	Flow cytometry
☒	☐	MRI-based neuroimaging

Antibodies

Antibodies used	Immunohistochemistry on mouse tissue: nucleoporin p62 (BD Biosciences 610497, ms 1:400); anti-GFP (Millipore Sigma AB16901, chk 1:2500); anti-NeuN (Cell Signaling Technology 24307, rb 1:200), Alexa Fluor 647 goat-anti-mouse (Thermo Fisher A32728), Alexa Fluor 546 donkey-anti-rabbit (Thermo Fisher A10040), and Alexa Fluor 488 goat-anti-chicken (Thermo Fisher A11039). Immuno histochemistry on post-mortem tissue: Nup62 (BD Biosciences, 610497, 1:100), NUP54 (Sigma-Aldrich, Cat. No. HPA-035929, 1:200), NUP98 (Abcam, Cat. No. ab50610, 1:250), pTDP43 1D3 (Millipore Sigma, Cat. No. MABN14 orBiolegend, Cat. No. 829901), TDP-43 (Proteintech, 10782-2-AP), p62 (BD Transduction Laboratory, 610833), FUS (Sigma Aldrich, Cat. No. HPA008784) Immunocytochemistry: MAb414 (1:1000, Biolegend, Cat. No. 902901), NUP62 (1:400, Abcam, Cat. No. ab188413 or 1:500, Millipore, MABE1043), MAP2 (1:1000, Synaptic Systems, Cat. No. 188044), NUP153 (1:300, Abcam, Cat. No. ab84872), NUP54 (2 ug/mL, Abcam, ab220890), NUP98 (1:500, Abcam, ab50610), TDP-43 (1:200, Proteintech, Cat. No.12892-1-AP or 10782-2-AP), ATAXIN-2 (1:400, Proteintech, Cat. No. 21776-1-AP), G3BP1 (1:300, Santa Cruz, Cat. No. sc-365338) Donkey anti-Mouse (1:1000, Jackson Immunoresearch), Donkey anti-Rat (1:1000, Jackson Immunoresearch), Donkey anti-Guinea Pig (1:1000, Jackson Immunoresearch), Donkey anti-Rabbit (1:1000, Jackson Immunoresearch).
Validation	All antibodies employed were previously validated by the manufacturing company.

Eukaryotic cell lines

Policy information about cell lines

Cell line source(s)	HEK293 cells (ATCC), induced pluripotent stem cells (Boulting et al., 2011; Nat Biotech, RUECDR Infinite Biologics, Coriell Institute for Medical Research, Cedars Sinai iPSC Core)

Authentication	These cell lines were authenticated by the laboratory or company of origin.
Mycoplasma contamination	Cell lines were not tested for mycoplasma contamination but no indication of mycoplasma contamination was observed.
Commonly misidentified lines (See ICLAC register)	This work did not include any commonly misidentified lines.

Animals and other organisms

Policy information about studies involving animals; ARRIVE guidelines recommended for reporting animal research

Laboratory animals	C57BL/6 mice, male, 12 months old
Wild animals	No wild animals were used in this study.
Field-collected samples	No field-collected samples were used in this study
Ethics oversight	All animal husbandry and procedures were carried out with the approval and oversight of the Thomas Jefferson University Institutional Animal Care and Use Committee (IACUC). Additionally, all procedures were carried out in compliance with the National Institutes of Health Guide for the Care and Use of Laboratory Animals. All animals were provided food and water ad libitum in a temperature, humidity, and light-controlled animal facility

Note that full information on the approval of the study protocol must also be provided in the manuscript.

Human research participants

Policy information about studies involving human research participants

Population characteristics	*Describe the covariate-relevant population characteristics of the human research participants (e.g. age, gender, genotypic information, past and current diagnosis and treatment categories). If you filled out the behavioural & social sciences study design questions and have nothing to add here, write "See above."*
Recruitment	*Describe how participants were recruited. Outline any potential self-selection bias or other biases that may be present and how these are likely to impact results.*
Ethics oversight	For use of human post-mortem tissue: Autopsy consent with research permission was provided by next-of-kin at time of death.

Note that full information on the approval of the study protocol must also be provided in the manuscript.
